# The Gender Gap in Second Language Acquisition: Gender Differences in the Acquisition of Dutch among Immigrants from 88 Countries with 49 Mother Tongues

**DOI:** 10.1371/journal.pone.0142056

**Published:** 2015-11-05

**Authors:** Frans W. P. van der Slik, Roeland W. N. M. van Hout, Job J. Schepens

**Affiliations:** 1 Department of Linguistics, Radboud University, Nijmegen, The Netherlands; 2 Max Planck Institute for Human Development, Berlin, Germany; Leiden University, NETHERLANDS

## Abstract

Gender differences were analyzed across countries of origin and continents, and across mother tongues and language families, using a large-scale database, containing information on 27,119 adult learners of Dutch as a second language. Female learners consistently outperformed male learners in speaking and writing proficiency in Dutch as a second language. This gender gap remained remarkably robust and constant when other learner characteristics were taken into account, such as education, age of arrival, length of residence and hours studying Dutch. For reading and listening skills in Dutch, no gender gap was found. In addition, we found a general gender by education effect for all four language skills in Dutch for speaking, writing, reading, and listening. Female language learners turned out to profit more from higher educational training than male learners do in adult second language acquisition. These findings do not seem to match nurture-oriented explanatory frameworks based for instance on a human capital approach or gender-specific acculturation processes. Rather, they seem to corroborate a nature-based, gene-environment correlational framework in which language proficiency being a genetically-influenced ability interacting with environmental factors such as motivation, orientation, education, and learner strategies that still mediate between endowment and acquiring language proficiency at an adult stage.

## Introduction

Contemporary handbooks on second language acquisition hardly pay attention to the role of learners’ gender (see [1–4]). A simple reason for the relative absence of research on the role of gender in L2 acquisition might be that female L2 learners doing better than male learners is regarded as being common knowledge. Perhaps Saville-Troike (2005: p.90) expresses the situation best when she critically notes: “There is widespread belief in many western cultures that females tend to be better L2 learners than males, but this belief is probably primarily a social construct, based on outcomes which reflect cultural and sociopsychological constraints and influences” [5].

The sparse research on the impact of gender in L2 acquisition shows a marked contrast with the massive literature on gender effects in first language acquisition (L1) research [6–9]. In a large meta-analytic study with more than 4 million students, Cole (1997) and Willingham and Cole (1997) concluded that female students retained their language advantage over a period of 30 years [6, 9]. Female students fared better in writing and language use (i.e. grammatical conventions, expression, spelling), while small but consistent effect sizes were found for reading and verbal reasoning. Lietz (2006), Rosén (2001), and Wagemaker (1996) observed the existence of a gender gap in many countries around the world, favoring women over men, regarding language abilities [10–12]. Gender differences in L1 acquisition have also been found to occur in the earliest stages of the life span. Girls develop communicative skills at a younger age than boys exhibiting larger vocabularies and using a larger variety of sentences [13–14].

What precisely is the state of affairs of the spare empirical support for a gender gap in L2 acquisition? Burstall (1975) and Davies (2004) observed lower attainment scores for British boys than for girls with respect to learning French [15–16]. Pae (2004) found that females outperformed males in reading comprehension among Korean English foreign language (EFL) learners [17]. Boyle (1987) observed that female Chinese students outperformed their male counterparts on a general English proficiency test [18]. In sum, the available evidence, though sparse, agrees with results found in first language acquisition, female language learners outperforming male learners.

Do gender differences require a nature or nurture explanatory scenario or both? The L1 gender distinction nicely matches other basic gender distinctions occurring early in the life span. Halpern (2002) and Kimura (1999) provide ample evidence that differences between male and female cognitive functioning can partly be explained by different hormonal configurations [19–20]. The production of male sex hormones from early childhood on in boys is assumed to be critical in this respect. As a result, masculinization of behavior and cognition occurs, causing a variety of differences between men and women in, for example, motor skills, spatial abilities, mathematical aptitude, perception, and verbal abilities (see [9]).

It is tempting to generalize such a nature-based scenario to L2 acquisition. Ullman (2005) proposes a declarative/procedural model for the description of memory systems [21]. According to Ullman (but see [22] for counterevidence, and [23] for a partial confirmation), women, more than men, store verbal information in declarative memory, whereas men more than women tend to use grammatical or procedural memory to produce language and these processes are found to be accommodated by estrogen [24–25]. This distinction in the prevalence of different memory systems in men and women seems to be relevant for second language learning by adult learners as the availability of the procedural memory decreases more than declarative memory after puberty. As a result, adult female second language learners would be in a more advantageous position than adult male learners, although it is not clear in which language domains a female advantage would occur in particular. Based on the foregoing, it might be hypothesized that, on average, women will perform better than men on speaking and writing proficiencies, because productive skills require active access to all verbal and verbal-related resources available. When gender differences are present because language proficiency is a genetically influenced ability, the probability of these differences showing up seems to be best when this ability has to perform maximally, exploiting all resources.

The expected outcomes concerning listening and reading skills are not straightforward, judging from the sparse available empirical evidence. Farhady (1982) found a female advantage in listening [26], Boyle (1987) found a male advantage in listening vocabulary for Chinese EFL learners [18], but see [27], whereas Bacon (1992) detected no significant difference between Spanish male and female performance in listening skills [28].

An additional complicating factor is that the four modalities of speaking, writing, reading and listening are being tested with different testing formats. The format may affect the gender outcomes however. Walstad and Robson (1997) found that females perform worse on multiple-choice tests [29], whereas Lumsden and Scott (1987) observed that women performed better on open format tests [30], a format typically used for the elicitation of speaking and writing. Listening and reading are typically tested through multiple-choice tests, which seems to be disadvantageous for females.

An additional explanation for the lack of unequivocal evidence of a gender gap in L2 acquisition is that genes and environment (nature and nurture) interact in complex ways and on levels ranging from the individual to the societal [31–34]. Indeed, in some countries a supposed genetic advantage of women with respect to second language acquisition might be obscured by, for example, unequal schooling opportunities or different gender roles that put women in a disadvantageous position. However, in the United States a trend was observed of increased female participation in tertiary schooling and from the early 1980s onwards women’s participation has surpassed men’s [35]. Moreover, in a vast majority of other Western countries, too, the female-to-male ratio in tertiary education presently exceeds one [36]. There seems to be a trend that, at least in Western countries, and with respect to tertiary education, women’s disadvantaged position in society is disappearing. As a consequence, the female advantage in second language learning might be better observable in L2 learners originating from Western countries. Correlations between gene and environment related factors [34, 37–38] might enforce or mitigate the way genes and schooling interact in diverse causality schemes. An extensive elaboration of these schemes is beyond the scope of the present study, given the non-experimental data we have investigated. Some explanatory schemes will be touched upon in the discussion.

Apart from societal changes and the amount of variation at the macro-level between countries and languages, some recent L2 theories put special emphasis on the enormous complexity of L2 acquisition. Larsen-Freeman (1997) and De Bot, Lowie, and Verspoor (2007), for example, use the concept of Dynamic Systems Theory to disclose how small distinctions in initial conditions and the way how various subsystems interact over time both within and between subjects, might bring about large differences in L2 proficiency in a non-linear fashion [39–40]. However, acknowledging the complexity of the L2 learning process does not reduce the relevance of gender-related differences. That is illustrated by studies that try to put forward more concrete explanatory factors, as in observing and investigating learning strategies. The general pattern that emerges is that women report a more frequent use of cognitive and meta-cognitive learning strategies than men [41–44]. In addition, instigated by the work of Gardner [45], many studies have concentrated upon motivational aspects of learning an additional language. There are good reasons for such an interest, because as Cohen and Dörnyei (2002: p.172) put it: “Motivation is often seen as the key learner variable because without it, nothing much happens” [46]. Several studies have observed that women are more motivated than men in L2 learning [2, 47–48]. At the same time, women have been found to have more positive attitudes toward studying a foreign language than male language learners [49–50], and to have more interest in a target culture [51]. It is, thus, evident that factors which have presumably a nurture origin play an enforcing role in shaping a gender gap in L2.

Given what is known about gender differences in L2 acquisition, we first want to answer the question whether there is a consistent, robust gap between men and women in adult L2 acquisition. We use a large database of L2 Dutch to provide clear empirical support for a general L2 gender gap. The database contains proficiency scores on speaking and writing, tapping the productive part of language proficiency, and on reading and listening, testing the receptive part of language proficiency. Including these four modalities may give a rather comprehensive insight into factors that play a role in establishing gender differences. Our aim is to establish explanatory scenarios that can account for the differences we found in language proficiency between learners with different nationality and language backgrounds. Such a scenario needs to include interaction effects of gender with other characteristics, be it on the individual or contextual level.

If there is a robust, consistent gender effect (across counties and languages), and assuming it has a nature-based, genetically-influenced foundation we expect that it may interact with environmental, nurture factors, increasing the gap the longer or stronger these external factors can do their work. It implies that female learners would accumulate their advantage over time (accumulating advantage is known as the Matthew effect in sociology [52]). Interaction effects may provide additional circumstantial evidence for the claim that women’s advantage regarding second language proficiency has primarily a start in nature and that its origin has no educational, socio-cultural or socio-economic foundation.

## The Present Study

We made use of a large-scale database, containing information on 27,119 migrants to examine the impact of gender on their proficiency scores in L2 Dutch. The migrants did an official test which yielded results on direct measures of speaking, writing, reading, and listening proficiency in Dutch. This approach has not been adopted in the past on such a massive scale and it allowed us to test the impact of gender along and in combination with a variety of other potential confounding variables. In this study, we wanted to flesh out how consistent and robust the gender effect is in L2 acquisition. The large-scale data base that we used provides a particular strong testing ground given the huge variety of L1s and countries of origin and given the social-demographic and contextual characteristics we could include in the statistical analyses.

We examined the impact of gender in speakers of 49 mother tongues, spoken in 88 countries, on the acquisition of Dutch as a target language. 27 mother tongues were Indo-European (IE) and 22 were non-Indo-European (non-IE). In the latter group, there are three Niger-Congo languages, five Afro-Asiatic, three Austronesian, and three Uralic languages. There was one Altaic (Turkish), one Dravidian (Tamil), one Kartvelian (Georgian), one Austro-Asiatic (Vietnamese), one Japanese, one Korean, one Sino-Tibetan (Chinese), and one Tai-Kadai (Thai) language. Examinees originated from 38 Western countries (including Canada, the United States, South Africa, and former East European countries), 16 African countries (South Africa excluded), 14 countries from South and Central America, 10 from East Asia, seven from the Middle East, and three South Asian counties. Countries of origin had to contain at least 20 examinees in order to be included in this study.

## Method

### Sample

Since the early 1990s, the State Examination of Dutch as a Second Language (STEX) is administered three times each year. These examinations consist of two separate exams. Program II (STEX II) is offered to immigrants who intend to enroll in a higher-level education in the Netherlands, or who have a higher-level occupation. Program I (STEX I) is aimed at immigrants who intend to follow a lower level of (vocational) education, or who have a lower or middle-level occupation. The requirements for Dutch language proficiency are the same for both levels, though the abstraction level for Program II is higher (for detailed information, see [53–54]). It is perhaps important to note that when taking lessons in Dutch, learners are given the opportunity to test their acquired level of proficiency by means of older state exams.

Test results were available from the Program II exams from the years 1995 up to 2004. The examination covers four language skills: speaking, writing, reading, and listening, which are tested separately. An examinee passes the entire exam when he or she has acquired 500 points or more on each of the four sub-exams. In the current study, the productive skills of speaking and writing proficiency in Dutch as well as their receptive counterparts, reading and listening skills, have been analyzed. In total, 29,767 examinees took at least one of the four sub-exams in the period 1995–2004. In case of re-exams, we only used the first available test score. Data on test scores, gender and age were available for all examinees, as based on administrative data. Only adult second language learners between 18 and 50 years of age were included in the study.

Before the actual examination took place, examinees were invited to return a brief questionnaire about various background characteristics, such as date of arrival in the Netherlands, country of birth, mother tongue, education, etc., that was sent to them when they subscribed for the exam.

### Variables

In total, test scores of 27,119 language learners were available who had valid scores on at least one of the four language tests. We have chosen to opt for this inclusive approach, because selecting only language learners who took all four tests would substantially increase the probability of systematically excluding less successful learners, and, thus, underestimating learning effects. All variables involved are summarized in Table 1.

**Table 1:** Description of the Sample for the Dependent Variable, the Dutch Speaking, Writing, Reading, and Listening Proficiency Test, and the Explanatory Variables, Split out between Learner and Context Characteristics (49 L1s and 88 Countries), for Male and Fmale Learners.

	*Males*		*Females*		
	*Mean*	*SD*	*Mean*	*SD*	*n*
*Criterion*					
Dutch Speaking Proficiency Test	501	38	522	38	26,084
Dutch Writing Proficiency Test	507	47	535	46	26,383
Dutch Reading Proficiency Test	506	37	524	40	26,852
Dutch Listening Proficiency Test	496	39	516	43	26,667
*Learner Characteristics*					
Age of Arrival in the Netherlands	27.59	6.07	27.21	6.06	27,119
Length of Residence (at first exam date)	3.45	3.32	3.11	3.33	27,119
Number of Hours Studying Dutch language / 100	5.62	4.46	5.41	4.35	27,119
Years of Education	8.37	2.72	8.19	2.84	27,119
Western Country	.30	.46	.65	.48	14,090
Middle East	.31	.46	.10	.30	4,830
South- and Middle America	.03	.18	.07	.26	1,573
Africa	.26	.44	.06	.24	3,697
South Asia	.06	.23	.02	.14	851
East Asia	.04	.19	.10	.29	2,078
*Context Characteristics*					
Morphological Linguistic Distance	.07	.08			49
Gross Enrolment in Secondary Schooling in 1995	72.24	34.48			88

#### The criterion variables: Speaking, writing, reading, and listening test scores in Dutch

The Dutch proficiency tests, speaking, writing, reading and listening were constructed by the Centraal Instituut Toetsontwikkeling (Central Institute for Test Development) and the Bureau Interculturele Evaluatie (Bureau for Intercultural Evaluation)–two large test battery constructors in the Netherlands.

*Dutch Speaking Proficiency Test*. The typical speaking test consists of 14 assignments. The candidates are urged to respond orally to prompts like: “*In Dutch television a lot of ads are made for all kinds of products*, *even in the middle of a program*. *What is your opinion about ads on TV*?*”* These spoken elicitations were recorded on tape. The examination took 30 minutes. Two independent expert raters evaluated the oral production on content and correctness criteria. The primary content criteria are the appropriateness of the content related to the task (about 30%) and vocabulary size (around 18%). The most important linguistic criteria are word and sentence formation (about 28%), and pronunciation (about 12%) (for detailed information, see [51–52]). Candidates can speak freely, but their responses are evaluated only when their responses go with the speech task.

*Dutch Writing Proficiency Test*. The writing test lasts 60 minutes, and a typical writing test consists of three different tasks: writing eight short responses on prompts, writing two short texts, and one longer text between 150 and 300 words. Two independent expert raters evaluated the written production on content and correctness. The primary content criterion is adequacy/understandability (about 40%). The most important linguistic criterion is grammatical correctness (about 30%) (for detailed information, see [55–56]).

It has to be emphasized that for both candidate’s speaking and writing production, the expert raters are bound to well-elaborated coding schemes that leave little room for subjective evaluations. Apart from that, all expert raters are experienced language teachers who, without exception, have undergone extensive scoring training. A candidate’s score is the mean of the two rater scores, which reduces potential bias even further. In case these two raters disagree about the passing or failing of a candidate, a third rater reviews the candidate’s writing or speaking production. The rater deviating most from the third one will be discarded in the final decision about a candidate’s test score.

*Dutch Reading Proficiency Test*. The reading test lasts 20 minutes and examinees have to read nine in length varying texts on a variety of subjects (i.e. instructions of a dental surgeon; study regulations), and answer in total around 53 multiple choice questions.

*Dutch Listening Proficiency Test* Examinees have to listen to six recorded interviews in the listening task. These recorded interviews were played back in an examination room for all examinees simultaneously. No headphones were used. After hearing each interview they have to answer a number of multiple choice questions. The skills to be evaluated are global and selective listening based on oral reports and opinions. This examination takes 60 minutes and candidates have to answer a total of 41 multiple choice questions.

The difficulty of the examinations was held constant over time, by applying a specific Item Response Theory (IRT) model, namely the One-Parameter Logistic Model [57]–an advanced type of Rasch model. A decisive advantage of IRT models as compared to models based on Classical Test Theory is that the test scores of candidates who took the exam on different occasions are allocated to the same ability distribution; hence their test results can be analyzed simultaneously. In order to do so, parts of older exams were used in new exams (though the actual design was much more complex). The scores on the exam were standardized; 500 marks or more implied that the candidate had passed the exam and indicates that an examinee has a proficiency at the B2 level (independent user, vantage level) as defined in the Common European Framework [58], equivalent to the *International English Testing System* (IELTS 5.5) [59].

#### Learner characteristics

*Gender*. Females were coded as 1, males as 0.

*Age of arrival in the Netherlands*. On the basis of information on ‘Year of birth’ and ‘Date of arrival’, age at the time examinees arrived in the Netherlands was calculated.

*Length of residence*. On the basis of information on ‘Date of the exam’ and ‘Date of arrival’, length of residence in the Netherlands was calculated.

*Number of hours studying the Dutch language*. Examinees were also asked how many hours they typically studied Dutch in an average week, and for how many months they did this. Based on this information, the estimated number of hours that they used for studying the Dutch language was calculated.

*Years of education*. Examinees were asked if they received secondary and tertiary schooling, and if so, for how many years. Based on this information, the number of years of education was calculated. Examinees who provided valid information on their tertiary education but failed to do so on their secondary schooling were excluded from further analyses.

All variables were centered around their grand mean to reduce the risks of multicollinearity in interaction and higher order terms [60].

#### Context characteristics

*Linguistic distance*. Recently, a versatile measure of linguistic distance was developed which measures the morphological complexity of a first language relative to Dutch [56]. This measure was constructed on the basis of 29 morphological features of first languages that are used by Lupyan and Dale (2010) and documented in the WALS database [61–62]. This measure of decreasing morphological complexity showed that, as was predicted, the less morphologically complex a first language is in comparison to Dutch, the lower the scores of adult learners of Dutch are.

*Educational accessibility*. The World Bank [63] reports on education data in a huge number of countries around the world on a regular basis. We took the gross enrolment rate in secondary schooling per country in 1995 as an indicator for a country’s educational accessibility.

*Geographic region*. In order to capture potential gender differences in larger regions of countries of origin [64], we made a distinction between different geographic regions: Western countries, South and Central America, Africa, the Middle East, South Asia and East Asia. Western countries were used as the reference category.

In Table 1, the descriptive statistics of the sample are presented for male and female learners. For all tests, the ‘Dutch Speaking Proficiency Test’, the ‘Dutch Writing Test’, the ‘Dutch Reading Test’, and the ‘Dutch Listening Proficiency Test’, female learners’ scores turn out to be approximately half a standard deviation higher than male learners’ scores. Given the huge number of examinees studied here, these differences are clearly significant (*T*(26,082) = 43.84, *p* <. 0001, *T*(23,381) = 46.44, *p* < .0001, *T*(21,757.9) = 36.72, *p* < .0001,and *T*(220,61.3) = 37.61, *p* < .0001, respectively). These overall differences might mask substantive variation in the gender gap across mother tongues and countries of origin, however. For speaking proficiency, female learners were doing better in 67 out of 88 countries of which 16 effects were significant. None of the differences where men scored higher than women was significant. For writing, the outcomes are almost identical: again 67 out of 88 countries were in favor of females, 20 significantly; in only one country males performed significantly better than females: Cameroon. For reading and listening, an entirely different picture emerged. Female learners performed better on reading than male learners in only 32 countries, just three differences being significant (Algeria, Morocco, Turkey), while in eight countries male learners outperformed female learners significantly (see, S1, S2, S3 and S4 Tables for detailed information). For listening skills, the trend was in the direction of females outperforming males in 46 countries and in six countries these differences were actually significant—China being one of them. In the remaining 42 countries, males were doing better and in three countries (Afghanistan, Czech Republic and Vietnam) this difference was actually significant. There seems to be a marked distinction between productive and receptive second language abilities but further analyses of these varying outcomes is needed to prevent whatever speculation about their interpretation.

### Analyses

We used cross-classified multilevel models (we used SPSS 21 [65]), to take full account of the multilingual reality, i.e. migrants from different countries may use the same mother tongue, while migrants from the same country may speak different first languages. In doing so, we were able to test for gender interaction effects, if present, at the learner level, the mother tongue level, and the country of origin level.

We first constructed null models for the four Dutch language proficiency tests separately with no predictive factors added. Learners, countries and mother tongues were included as random factors (random intercepts). Next, we added gender to these null models as a baseline model (Model 1). Then, in Model 2, we added the remaining learner characteristics in order to test if the gender effect still holds. Next, we tested if the gender effect remained intact by adding interaction effects of gender with these learner characteristics in Model 3. Then, we added the country characteristics in Models 4 and 5. And finally, in Models 6 and 7, language characteristics were included.

The improvement in fit signifies that the model fits the data better and this is tested by means of the Log Likelihood ratio which follows a Chi-square distribution. A given model is considered to have a better fit than a preceding, more parsimonious model if the difference in the Log Likelihood ratio (–2L^2^) is at least 3.84 against one degree of freedom. When the improvement of fit of the less parsimonious model as compared with the previous model was significant, we checked the direction and size of the effect parameters.

The application of the null model to the proficiency scores resulted in three random variance components (so-called intra-unit correlations). They showed that, for speaking proficiency, 14.6% of the variation in scores is across languages and 15.6% across countries. Summing these up (see e.g. [66]), we observed that 30.2% of the total variance is to be attributed to country and language characteristics. For writing proficiency, the distribution is 11.4% across countries; 14.7% across languages (total: 26.1%), while for reading proficiency 15.3% of the variation in scores is across countries and 20.6% across languages (35.9%). For listening proficiency, finally, the variation in scores across languages was 23.7% and across countries12.8%, giving a total of 36.5% of the total variance. Accordingly, the remaining variance at the individual level was estimated at 69.8%, 75.9%, 64.1%, and 65.5% of the total variance in respectively speaking, writing, reading, and listening proficiency scores.

In the next step, we added fixed level 1 explanatory variables to the cross-classified design of languages by countries for speaking (see Table 2), writing (see Table 3), reading (Table 4) and listening (Table 5) proficiency in Dutch. Given the huge amount of data used in this study, effects were tested at the alpha = .01 level.

**Table 2:** Cross-classified Multilevel Model Parameter Estimations for Measures of the Dutch Speaking Proficiency Test (Standard Errors in Parentheses) per Mother Tongue and Country of Birth.

	Model 1	Model 2	Model 3	Model 4	Model 5	Model 6	Model 7 Final Model
Intercept	512.55*** (2.78)	512.77*** (2.86)	512.78*** (2.86)	510.13*** (1.98)	514.39*** (1.98)	513.23*** (1.91)	513.30*** (1.94)
Female	7.87*** (.48)	7.70*** (.47)	7.71*** (.47)	7.66*** (.47)	7.52*** (.49)	7.52*** (.49)	7.99*** (.59)
Length ofResidence (LoR)		1.04*** (.06)	1.04*** (.06)	1.04*** (.06)	1.04*** (.06)	1.04*** (.06)	1.04*** (.06)
Age of Arrival(AoA)		–1.00*** (.04)	–.99*** (.04)	–.98*** (.04)	–.98*** (.04)	–.98*** (.04)	–.98*** (.04)
Number of HoursDutch (Lessons)		–.47*** (.05)	–.47*** (.05)	–.47*** (.05)	–.47*** (.05)	–.47*** (.05)	–.47*** (.05)
Years ofEducation		.95*** (.08)	.93*** (.08)	.93*** (.08)	.93*** (.08)	.93*** (.08)	.93*** (.08)
Female*LoR			–.15 (.12)	–.16 (.12)	–.21 (.13)	–.21 (.13)	–.21 (.13)
Female*AoA			–.09 (.07)	–.09 (.07)	–.05 (.07)	–.05 (.07)	–.05 (.07)
Female*Lessons			–.16 (.09)	–.16 (.10)	–.20 (.10)	–.20 (.10)	–.22* (.10)
Female*Education			.74*** (.16)	.74*** (.16)	.68*** (.16)	.68*** (.16)	.71*** (.16)
Gross Enrolment				.17* (.07)	.17* (.07)	.16* (.06)	.16* (.06)
Africa				–19.99***(6.11)	–19.11*** (6.11)	–18.75*** (5.72)	–18.68** (5.75)
Middle East				–9.30 (5.50)	–9.52 (5.50)	–9.83 (5.12)	–9.61 (5.18)
East Asia				–22.36*** (5.34)	–24.00*** (5.35)	–11.44 (6.50)	–11.61 (6.58)
South Asia				–12.71 (7.91)	–13.60 (7.90)	–13.38 (7.35)	–13.04 (7.44)
Central and South America				.10 (6.65)	.78 (6.64)	–.33 (6.19)	–.11 (6.27)
Female*Gross Enrolment					–.00 (.03)	–.00 (.03)	–.01 (.03)
Female*Africa					5.21* (2.36)	5.11* (2.36)	4.89* (2.37)
Female*Middle East					2.09 (1.68)	2.05 (1.68)	1.73 (1.70)
Female*East Asia					7.20** (2.33)	7.08** (2.33)	3.41 (3.50)
Female*South Asia					–2.53 (3.22)	–2.55 (3.22)	–2.58 (3.22)
Female*Central and South America					–4.87* (2.47)	–4.91* (2.47)	–4.60 (2.48)
Morphological Complexity						-90.14** (29.69)	-86.86** (30.45)
Female* Morphological Complexity							23.74 (16.84)
Residual	1063.83*** (9.33)	1016.52*** (8.92)	1015.55*** (8.91)	1015.90*** (8.92)	1014.71*** (8.91)	1014.91*** (8.91)	1014.79*** (8.91)
Variance Mother Tongue	225.85** (79.21)	245.32** (82.97)	245.60** (83.57)	105.22** (33.13)	104.90** (33.19)	98.92** (32.37)	102.78** (33.60)
Variance Country	192.67*** (45.32)	193.00*** (45.02)	193.79*** 45.35)	78.34*** (20.01)	77.87*** (20.05)	62.99*** (17.62)	64.43*** (18.00)
-2 Log Likelihood	256,166	254,998	254,980	254,882	254,836	254,819	254,809

*: *p* < .05

**: *p* < .01

***: *p* < .001

**Table 3:** Cross-classified Multilevel Model Parameter Estimations for Measures of the Dutch Writing Proficiency Test (Standard Errors in Parentheses) per Mother Tongue and Country of Birth.

	Model 1	Model 2	Model 3	Model 4	Model 5	Model 6	Model 7 Final Model
Intercept	524.42*** (3.14)	525.34*** (3.13)	525.34*** (3.13)	525.58*** (2.31)	525.58*** (2.32)	524.58*** (2.32)	524.52*** (2.30)
Female	9.05*** (.60)	9.14*** (.59)	9.13*** (.59)	9.05*** (.59)	9.16*** (.61)	9.15*** (.61)	8.39*** (.73)
Length of Residence (LoR)		–.42*** (.08)	–.41*** (.08)	–.41*** (.08)	–.41*** (.08)	–.41*** (.08)	–.41*** (.08)
Age of Arrival (AoA)		–.77*** (.04)	–.76*** (.04)	–.75*** (.04)	–.76*** (.04)	–.76*** (.04)	–.76*** (.04)
Number of Hours Dutch (Lessons)		–.36*** (.06)	–.36*** (.06)	–.36*** (.06)	–.36*** (.06)	–.36*** (.06)	–.36*** (.06)
Years of Education		2.64*** (.10)	2.62*** (.10)	2.61*** (.10)	2.62*** (.11)	2.62*** (.10)	2.62*** (.10)
Female* LoR			–.10 (.15)	–.09 (.15)	.09 (.15)	–.09 (.15)	–.10 (.16)
Female*AoA			–.26** (.09)	–.26** (.09)	–.23** (.09)	–.23** (.09)	–.23* (.09)
Female*Lessons			–.27* (.12)	–.27* (.12)	–.24* (.12)	–.24* (.12)	–.21* (.12)
Female*Education			.76*** (.20)	.76*** (.20)	.77*** (.20)	.77*** (.20)	.72*** (.20)
Gross Enrolment				.14* (.07)	.15* (.07)	.14* (.06)	.13* (.06)
Africa				–23.92*** (6.18)	–23.16*** (6.26)	–22.79*** (6.00)	–22.80*** (5.95)
Middle East				–17.93** (5.57)	–17.57** (5.63)	–17.56** (5.40)	–17.71** (5.35)
East Asia				–17.83** (5.88)	–18.70** (5.94)	–7.59 (7.45)	–7.58 (7.40)
South Asia				–14.00 (8.07)	–15.42 (8.15)	–15.24 (7.82)	–15.54 (7.76)
Central and South America				–7.90 (6.69)	–7.51 (6.77)	–8.10 (6.46)	–8.30 (6.40)
Female*Gross Enrolment					–.00 (.04)	–.00 (.04)	–.02 (.04)
Female*Africa					1.49 (2.93)	1.43 (2.93)	1.79 (2.93)
Female*Middle East					–.03 (2.08)	–.05 (2.08)	.46 (2.10)
Female*East Asia					3.57 (2.90)	3.49 (2.90)	9.57* (4.34)
Female*South Asia					–12.06** (3.99)	–12.04** (3.99)	–12.00** (3.99)
Female*Central and South America					–1.06 (3.08)	–1.68 (3.08)	–2.21 (3.09)
Morphological Complexity						–85.18* (36.68)	–88.76* (36.51)
Female* Morphological Complexity							–39.08 (20.73)
Residual	1635.29*** (14.27)	1579.78*** (13.78)	1578.44*** (13.77)	1578.93*** (13.78)	1577.95*** (13.77)	1578.08*** (13.77)	1578.02*** (13.77)
Variance Mother Tongue	305.22** (98.34)	314.20** (100.11)	314.83** (100.74)	179.99*** (53.44)	179.85*** (53.59)	179.20*** (52.10)	177.20*** (51.31)
Variance Country	213.53*** (51.24)	197.83*** (48.50)	197.89*** (48.73)	57.77** (19.24)	59.87** (19.66)	51.21** (17.37)	49.82** (16.49)
-2 Log Likelihood	270,418	269,517	269,499	269,398	269,364	269,351	269,339

*: *p* < .05

**: *p* < .01

***: *p* < .001

**Table 4:** Cross-classified Multilevel Model Parameter Estimations for Measures of the Dutch Reading Proficiency Test (Standard Errors in Parentheses) per Mother Tongue and Country of Birth.

	Model 1	Model 2	Model 3	Model 4	Model 5	Model 6	Model 7 Final Model
Intercept	517.93*** (3.18)	518.90*** (3.18)	518.92*** (3.18)	519.15*** (2.38)	519.61*** (2.39)	518.59*** (2.38)	518.57*** (2.37)
Female	–1.44*** (.45)	–1.07* (.44)	–1.07* (.44)	–1.12** (.44)	–1.44*** (.45)	–1.44*** (.45)	–1.73** (.55)
Length of Residence (LoR)		–.25*** (.06)	–.24*** (.06)	–.24*** (.06)	–.24*** (.06)	–.24*** (.06)	–.24*** (.06)
Age of Arrival (AoA)		–.76*** (.03)	–.75*** (.03)	–.74*** (.03)	–.74*** (.03)	–.74*** (.03)	–.74*** (.03)
Number of Hours Dutch (Lessons)		–.72*** (.04)	–.72*** (.04)	–.71*** (.04)	–.72*** (.04)	–.72*** (.04)	–.72*** (.04)
Years of Education		2.76*** (.08)	2.73*** (.08)	2.73*** (.08)	2.73*** (.08)	2.73*** (.08)	2.73*** (.08)
Female* LoR			.02 (.11)	.02 (.11)	–.06 (.12)	–.06 (.12)	–.06 (.12)
Female*AoA			–.30*** (.07)	–.30*** (.07)	–.26*** (.07)	–.26*** (.07)	–.26*** (.07)
Female*Lessons			–.19* (.08)	–.19* (.08)	–.29*** (.09)	–.28*** (.09)	–.27** (.09)
Female*Education			1.12*** (.15)	1.11*** (.15)	1.02*** (.15)	1.02*** (.15)	1.00*** (.15)
Gross Enrolment				.19** (.07)	.19** (.07)	.18** (.06)	.18** (.06)
Africa				–21.88*** (6.15)	–21.19*** (6.17)	–20.93*** (5.92)	–20.91*** (5.90)
Middle East				–13.04** (5.46)	–13.01* (5.48)	–12.81* (5.27)	–12.85* (5.25)
East Asia				–12.96* (5.90)	–13.91* (5.93)	–2.18 (7.21)	–2.27 (7.19)
South Asia				–14.31 (7.98)	–14.99 (8.00)	–14.82 (7.69)	–14.94 (7.66)
Central and South America				–5.83 (6.83)	–6.38 (6.85)	–7.11 (6.56)	–7.11 (6.56)
Female*Gross Enrolment					–.00 (.03)	–.00 (.03)	–.01 (.03)
Female*Africa					6.20** (2.18)	6.15** (2.18)	6.29** (2.18)
Female*Middle East					4.67** (1.55)	4.66** (1.55)	4.85** (1.56)
Female*East Asia					4.66* (2.15)	4.61* (2.15)	6.88* (3.24)
Female*South Asia					1.09 (2.97)	1.10 (2.97)	1.11 (2.97)
Female*Central and South America					3.22 (2.29)	3.21 (2.29)	3.01 (2.30)
Morphological Complexity						–96.18** (36.33)	–97.29** (36.20)
Female* Morphological Complexity							–14.67 (15.59)
Residual	957.76*** (8.28)	891.42*** (7.71)	889.15*** (7.69)	889.19*** (7.69)	888.21*** (7.68)	888.22*** (7.68)	888.25*** (7.68)
Variance Mother Tongue	310.23*** (96.41)	321.30*** (99.50)	322.62*** (100.43)	198.69*** (55.09)	199.20*** (55.41)	197.48*** (53.18)	195.25*** (52.61)
Variance Country	234.43*** (50.92)	218.98*** (48.21)	219.53*** (48.58)	65.33*** (16.74)	65.85*** (16.84)	57.79*** (14.65)	57.44*** (14.55)
-2 Log Likelihood	260,912	259,002	258,941	258,830	258,786	258,770	258,762

*: *p* < .05

**: *p* < .01

***: *p* < .001

**Table 5:** Cross-Classified Multilevel Model Parameter Estimations for Measures of the Dutch Listening Proficiency Test (Standard Errors in Parentheses) per Mother Tongue and Country of Birth.

	Model 1	Model 2	Model 3	Model 4	Model 5	Model 6	Model 7 Final Model
Intercept	507.72*** (3.42)	508.47*** (3.48)	508.49*** (3.48)	509.56*** (2.55)	509.77*** (2.56)	508.89*** (2.55)	508.87*** (2.54)
Female	.54 (.48)	.79 (.47)	.79 (.47)	.74 (.47)	.72 (.49)	.71 (.49)	.45 (.59)
Length of Residence (LoR)		.89*** (.06)	.89*** (.06)	.90*** (.06)	.90*** (.06)	.90*** (.06)	.90*** (.06)
Age of Arrival (AoA)		–.74*** (.04)	–.73*** (.04)	–.73*** (.04)	–.73*** (.04)	–.73*** (.04)	–.73*** (.04)
Number of Hours Dutch (Lessons)		–.74*** (.05)	–.74*** (.05)	–.74*** (.05)	–.74*** (.05)	–.74*** (.05)	–.74*** (.05)
Years of Education		2.14*** (.08)	2.11*** (.08)	2.11*** (.08)	2.11*** (.08)	2.11*** (.08)	2.11*** (.08)
Female* LoR			–.02 (.12)	–.03 (.12)	–.07 (.12)	–.07 (.12)	–.06 (.12)
Female*AoA			–.16* (.07)	–.17* (.07)	–.14* (.07)	–.14* (.07)	–.14 (.07)
Female*Lessons			–.19* (09)	–.19* (09)	–.23* (10)	–.23* (10)	–.22* (10)
Female*Education			1.10*** (.16)	1.10*** (.16)	1.06*** (.16)	1.07*** (.16)	1.05*** (.16)
Gross Enrolment				.19** (.07)	.19** (.07)	.18** (.07)	.18** (.07)
Africa				–21.32*** (6.42)	–20.57*** (6.45)	–20.35*** (6.24)	–20.34*** (6.22)
Middle East				–10.61 (5.71)	–10.67 (5.73)	–10.67 (5.54)	–10.73 (5.53)
East Asia				–21.05*** (6.25)	–22.07*** (6.29)	–12.32 (7.66)	–12.38 (7.63)
South Asia				–11.84 (8.34)	–12.72 (8.37)	–12.62 (8.10)	–12.74 (8.08)
Central and South America				–4.39 (7.11)	–4.46 (7.14)	–5.10 (6.89)	–5.18 (6.87)
Female*Gross Enrolment					–.02 (.03)	–.02 (.03)	–.02 (.03)
Female*Africa					3.04 (2.34)	3.00 (2.34)	3.12 (2.35)
Female*Middle East					.96 (1.67)	.95 (1.67)	1.12 (1.68)
Female*East Asia					4.14 (2.32)	4.10 (2.32)	6.16 (3.49)
Female*South Asia					–5.08 (3.20)	–5.07 (3.20)	–5.06 (3.20)
Female*Central and South America					–.30 (2.46)	–.30 (2.46)	–.49 (2.47)
Morphological Complexity						–82.16* (38.89)	83.34* (38.78)
Female* Morphological Complexity							–13.28 (16.85)
Residual	1076.16*** (9.34)	1021.81*** (8.87)	1019.79*** (8.85)	1019.98*** (8.85)	1019.48*** (8.85)	1019.56*** (8.85)	1019.60*** (8.85)
Variance Mother tongue	400.90*** (114.69)	421.40*** (120.52)	423.45*** (121.52)	233.26*** (61.49)	234.99*** (61.98)	229.65*** (59.79)	227.64*** (59.36)
Variance Country	213.75*** (47.93)	211.72*** (47.71)	211.82*** (47.91)	68.61*** (18.59)	68.99*** (18.65)	62.44*** (17.03)	62.04*** (16.94)
-2 Log Likelihood	262,217	260,853	260,808	260,702	260,674	260,661	260,653

*: *p* < .05

**: *p* < .01

***: *p* < .001

## Results

### Speaking Proficiency

We will first discuss the results on the speaking scores, presented in Table 2. For the outcomes of the writing, reading, and listening scores, only the final models will be discussed.

Initially, only Gender was added to the null model. This reduced the likelihood ratio with 262, against 1 degree of freedom which is highly significant. On average, women scored almost 8 points (*B* = 7.87, *SE* = .48, *p* < .001) higher than men did. Next, the remaining level 1 predictors were added (see Table 2, Model 2). The improvement in the likelihood ratio was 1,168 against 4 degrees of freedom. The effects of age of arrival (–1.00, *SE* = .04, *p* < .001), length of residence (1.04, *SE* = .06, *p* < .001), hours of studying Dutch (–.47, *SE* = .05, *p* < .001), and years of education (.95, *SE* = .08, *p* < .001) were all highly significant, but it is important to note that the effect of Gender (7.70, *SE* = .47, *p* < .001) remains largely unaffected by the inclusion of these potential confounding variables. The observed negative effect of number of hours studying Dutch may at first sight seem surprising. Remember, however, that during lessons examinees once in a while take probe exams in order to test their proficiency in Dutch. As lessons are in most cases not for free it seems safe to assume that examinees quit taking lessons once they expect to pass the actual exam, a decision not made so easily by less proficient language learners.

Next, interaction terms of gender with age of arrival, length of residence, hours studying Dutch, and years of education were added to the model (Model 3). The improvement in model fit was 187 points, against 4 degrees of freedom. It proved that only the interaction effect of gender with years of education (.74, *SE* = .16, *p* < .001) was significant while the interaction effects of gender with of age of arrival (–.09, *SE* = .07, *p* = .231), hours studying Dutch (–.16, *SE* = .09, *p* = .100), and with length of residence (–.15, *SE* = .12, *p* = .233) were not. The positive interaction effect with years of education seems to signify that ‘ceteris paribus’ women have benefited more from higher education than men did in learning Dutch. The higher the educational level the larger the differences between males and females, in favor of the females. Their gender advantage accumulates with an increasing educational level, the Matthew effect as we indicated earlier. In S1 Fig, we present a density plot of the raw scores (left panel) and the residuals of a model in which all the variables of the Final model are included except years of education, gender, and the interaction of gender and years of education (right panel). Darker colors represent higher density, blue representing male learners, pink female learners. The plot of residual scores, thus, describes the relationship of years of education and speaking skills in Dutch of male and female learners separately, but controlled for the effect of all other variables included in the model. The regression lines depicted in the right panel of S1 Fig represent the fixed γ_10_ effects of years of education for female and male learners separately. The values of the variable education were centered to the mean value. As a result, three years of secondary education corresponds with a value of –5.25, while 18 years of education equals 9.75. Applying the parameters of gender, education, and their interaction of the Final model, it can be calculated that the average gender gap when learners had received three years of secondary education is 4.26 points (*SE* = 1.00, *p* < .001) in favor of female learners (for males: .93 * -5.25 = -4.88; for females:7.99 + .71 * –5.25 + .93 * –5.25 = –.62), which is significant. However, in case they had 8.25 years of at least secondary education (the average number of years of education), this gap has widened to 7.99 points (*SE* = .59; *p* < .001).

In Model 4 of Table 2, country of origin characteristics have been included. The improvement of fit was significant with 98 against 6 degrees of freedom. The effect of educational accessibility as measured by gross enrolment in 1995 (.17, SE = .07, *p* = .012) turned out to be of borderline significance at the .01 level. There are indications that immigrants from countries characterized by a more developed (accessible) secondary schooling system perform better than immigrants from countries with a lesser developed (accessible) schooling system. Immigrants from African countries (*B* = –19.99, *SE* = 6.11, *p* = .002), and East Asia (*B* = –22.36, *SE* = 5.34, *p* < .001) performed worse than immigrants from Western countries, while speaking proficiency scores of immigrants originating from South Asia (*B* = –12.71, *SE* = 7.91, *p* = .113), the Middle East (*B* = –9.30, *SE* = 5.50, *p* = .095), and South and Central America (*B* = –.10, *SE* = 6.65, *p* = .988) did not differ significantly from those originating from Western countries. Inclusion of the interaction terms of gender with these country level characteristics, Model 5, resulted in a significant reduction of the deviance with 46 points against 6 degrees of freedom. Inspection of these interaction terms led to the conclusion that only the interaction effect of East Asia with Gender resulted in a significant interaction effect (*B* = 7.20, *SE* = 2.33, *p* = .002), indicating that, when taking their individual learner characteristics into account, East Asian female adult learners scored on average 7 points higher than East Asian male learners. The inclusion of the language context characteristic of morphological complexity, Model 6, resulted in a reduction of the deviance with 17 against one degree of freedom. Immigrants with mother tongues that are morphologically less complex linguistically than Dutch had lower scores on speaking skills than immigrants whose first languages are equally or more complex morphologically (*B* = –90.14, *SE* = 29.69, *p* = .004). Finally, the interaction term of gender with morphological complexity was included, Model 7. The improvement of fit was 10 against one degree of freedom. However, the interaction term of gender with morphological complexity turned out to be non-significant (23.74, *SE* = 16.84, *p* = .159). It can also be observed that the interaction effect of gender and originating from East Asia that was significant at the alpha = .01 level in previous models, has now become non-significant (4.89, *SE* = 2.37, *p* = .330). It is also important to note that the initial observed advantage of female immigrants over male immigrants with regard to Dutch speaking proficiency has endured: on average, female immigrants score 8 points higher on the speaking proficiency test (7.99, SE = .59, *p* < .001). This difference is found when all variables in the model have their mean value. Summing up, we found interaction effects where gender was one of the variables involved, but, without exception, these effects pointed in the direction of an increased female advantage. One of these effects is significant at *p* < .01 in Model 7, i.e. the interaction between education and gender.

### Writing Proficiency

In Table 3, the results for writing proficiency are presented. As announced in the previous section, only the Final model, Model 7 will be discussed. These outcomes for writing skills in Dutch corroborate those of speaking proficiency to a large extent. Female language learners scored on average more than eight points higher than male language learners, even when all investigated confounds and their interactions were taken into account. Again, the interaction of gender and years of education was significant (*B =* .72, *SE* = .20, *p* < .001) implying that ‘ceteris paribus’ female language learners benefited more from higher education than male learners did. The higher the educational level, the larger the difference between males and females become, in favor of the females, again pointing to a Matthew effect. Applying the parameters of gender, education, and their interaction in the final model, the average gender gap when learners had received three years of secondary education is 4.61 points (*SE* = 1.23, *p* < .001) in favor of female learners (for males: 2.62 * -5.25 = –13.76; for females: 8.39 + 2.62 * -5.25 + .72 * –5.25 = –9.15), which is significant. However, in case they had 8.25 years of at least secondary education (the average number of years of education), this gap has widened to 8.39 points (*SE* = .73; *p* < .001). See S2 Fig.

Finally, an interaction of region of origin and gender could be observed. Female learners from South Asia (Afghanistan, India, Pakistan, Sri Lanka) scored on average 12 points lower on the writing skills test than male South Asian learners. In sum, we found interaction effects where gender was one of the variables involved, but, again, these effects pointed in the direction of an increased female advantage.

### Reading Proficiency

The outcomes for reading proficiency depart from those of writing and speaking skills in Dutch fairly substantially. First of all, the gender effect in Model 7, the final model, is significantly negative (–1.73, *SE* = .55, *p* = .002), implying that males score on average almost two points higher than female language learners. Given the highly significant difference between male and female learners of Dutch overall the other way around, described earlier, this is a remarkable outcome. Though significant, this difference in reading skills can hardly be characterized as relevant, however, when interaction effects are taken into account. Several interaction effects with gender proved to be significant; the interaction between years of education and gender being one of them (1.00, SE = .15, p < .001), female language learners benefitting more from additional education than male language learners. The slope for female learners is steeper than for male learners. Applying a similar reasoning as for speaking proficiency, it can be deduced that male learners do not differ anymore from each other significantly when both sexes have had in between 9 and 12 years of education and the gender gap widens to 8.07 points (*SE* = 1.62; *p* < .001) in favor of women when female and male learners have reached their maximum level of education. See S3 Fig. The remaining interaction effects all point into the direction of a gender gap, favoring females over males. For example, female language learners from African countries, or from countries in the Middle East (Egypt, Iran, Iraq, Jordan, Kuwait, Lebanon, Syria, Turkey) respectively scored on average six and almost five points higher than their male counterparts (6.29, *SE* = 2.18, *p* = .004; 4.85, *SE* = 1.56, *p* = .002, respectively). We return to this in the Discussion.

### Listening Proficiency

We now turn to Table 5 for the effects on listening proficiency in Dutch. The effect of gender was not significant (*B* = .58, *SE* = .49, *p* = .232). Again, we found a positive interaction effect with years of education which seems to signify that ‘ceteris paribus’ women have benefited more from higher education than men did when learning Dutch. This interaction effect is similar to those found for speaking, writing and reading, but in this case without a general main gender effect. Applying a similar reasoning as for the three previous discussed proficiency tests, it can be deduced that male learners with three years of education score on average 5.06 points (*SE* = .99, *p* < .001) higher than female learners with a comparable level of education on listening proficiency; they do not differ from each other significantly when both sexes have had in between 5 and 9 years of education and the gender gap widens to 10.69 points (*SE* = 1.74; *p* < .001) in favor of women when female and male learners have reached their maximum level of education. See S4 Fig.

## Discussion and Conclusion

In the present study, our primary aim was to establish whether an overall gender difference exists in adult L2 acquisition, along the lines of the differences found in L1 acquisition, with females outperforming males. Our motive was the lack of clear empirical evidence for a L2 gender distinction. Research data are sparse, and the outcomes are not conclusive. We used a large database with test data from more than 25,000 adult learners of L2 Dutch from 88 countries of origin with 49 different mother tongues. We found a consistent gender effect for speaking and writing proficiency: Female learners outperformed male learners, independent of country of origin and mother tongue. This gender gap remained remarkably robust when individual, learner characteristics were taken into account, such as education, age of arrival, length of residence and number of lessons, or context characteristics, such as country of origin and mother tongue. The occurrence of these effects corroborate the validity of the gender gap found. These characteristics are known to have an impact on L2 proficiency. The negative role of number of lessons (hours studying Dutch), however surprising at first sight, could also be explained, because successful learners may stop attending lessons as soon as they believe to have reached the required level to pass the test. Perhaps the number of lessons works in a positive way at starting levels of language acquisition, but not any longer at higher levels of proficiency, as tested by the state exam Dutch as a second language (CEFR B2). For listening proficiency, this gender gap was absent and for reading proficiency it was even reversed: male language learners scored significantly higher on the reading in Dutch proficiency test than female language learners, although the difference between males’ and females’ reading proficiency scores was actually quite small.

The interaction effects we found for gender with other variables add in fact to the gender gap, strengthening it, as may be expected from a Matthew effect. The most remarkable effect is the interaction between gender and educational level, as measured by years of education. The gap between females and males widens with more years of education, signifying that females, ceteris paribus, profit more from more education. We found this increasing effect size for speaking, writing, reading, and listening proficiency. The interaction effects for reading and listening skills with years of education, however, cannot be qualified as a Matthew effect, as there is no starting or overall gender gap in favor of females. Because this interaction effect is returning in all four basic language skills, these increasing differences may be due to learner strategies, women being reported to use more frequently cognitive and meta-cognitive learning strategies than men [41–44] or to motivational distinctions [45–48], including perhaps attitudes toward studying a foreign language [49–50]. Such a scenario seems to point to intricate gene-environment interactions, but there is no reason to assume this scenario to be specific for second language skills. Even more reactive (evocative) genotype correlations [37–38] may be responsible, in part, for the observed differential effects of education, Language teachers may respond differently to male and female students in secondary education. Or, vice versa, students may respond differently to teachers. Perhaps, even recursive models are needed to interpret the origin and development of correlational patterns between gender, educational level and second language skills.

The observed gender gap for speaking skills could be argued to be the expression of expert raters’ unconscious bias favoring females over male examinees, even perhaps as a reactive genotype correlation reinforcing small gender differences. There are three reasons why such an explanation for the gender gap is fairly unlikely. First, as noted in the method section, the coding procedure used to rate examinees’ speaking production minimizes any such potential bias. Second, it might be assumed that the examinees’ gender is unknown in case of writing production, where nevertheless a gender gap is apparent as well. Finally, the Goldberg paradigm [67] predicts that females’ competences as compared to males’ will be downgraded not just by male raters but by female raters too. This paradigm still holds and is not just a relic from the past century, being too inaccurate to explain contemporary gender relationships [68].

The different outcomes for speaking and writing on the one hand, and reading and listening on the other, could be supposed to be due to test format. Females perform relatively worse on multiple choice tests than males [29], and relatively better on open format tests [30]. Reading and listening proficiency are typically tested through multiple choice tests, which is disadvantageous for females, and speaking and writing tests typically take the format of open questions, which is advantageous for females. When that is true, it means that the gender gap would be actually present as well in reading and listening, in fact enforcing our conclusion of a gender gap in adult second language acquisition. Given the enormous differences in language, geographical and educational (the different school systems) background of our learners, test format however cannot be expected to be the proper source to explain the distinction between these productive and receptive tests. This distinction seems at the same time to disqualify another explanatory scenario as being decisive. Several studies on motivational aspects concluded that women are more motivated than men in L2 learning [2, 47–48] and that women have more positive attitudes toward studying a foreign language than male language learners [49–50], and a stronger interest in the target culture [51]. Motivation, attitude and involvement cannot be considered conclusive sources to explain the gender distinction between productive and receptive language skills, however.

The gender distinction between productive (speaking, writing) and receptive (reading, listening) and the gender distinction present in acquisition (speaking, listening) and in learning (reading, writing) for that matter [69], seems to be a problem for any pure nurture, environment oriented scenario. Two nurture-driven theories on gender differences that could be relevant are the human capital approach, and gender-specific acculturation. The human capital framework bases gender differences in second language acquisition on the assumption that men participate more often than women in the labor process and the acquisition of the L2 is more important for them than it is for their wives, who in this approach are assumed to stay at home and take care of the children [70–71]. The outcomes of the present study seem to oppose any human capital approach. However, the human capital approach might also be used to explain immigrant men having a head start as compared to immigrant women due to pre-immigration characteristics. According to Beiser and Hou (2000), and Carliner (2000), South Asian men had higher proficiency levels in English than women [72, 64]. In our study we could replicate these findings in a rather rudimentary form for writing proficiency but not for speaking, listening and reading skills, however. It is relevant to note that the aforementioned studies, like most economic and sociological studies, make use of self-assessed measures of language proficiency. One important drawback of self-reported measures is that immigrants overstate their second language proficiency because they evaluate their skills relative to other immigrants [73]. In addition, Finnie and Meng (2005: p.1947) found that “women tend to underestimate the increases in literacy associated with higher levels of education relative to men” [73], which is presumed to be consistent with the notion that females generally tend to underestimate their capacities and talents [73].

The second nurture scenario takes gender-related acculturation patterns as determinants of differences between men and women in L2 acquisition [70, 74–75]. The scenario is that foreign and, in particular, second language acquisition, involves more than just mastering an additional language [76], because it also means “acquiring symbolic elements of a different ethnolinguistic community” ([77]: p.193). Learning the language of the dominant language community may thus be seen as a threat to one’s cultural identity and that very threat may affect the development of men’s and women’s second language proficiency differently. According to Polat and Mahalingappa (2010), a reason for immigrant women’s orientation on the dominant language might be that it enhances their social status and helps them to acquire more social and economic freedom [70]. However attractive such a line of argumentation might seem to be at first glance, we find it difficult to believe that immigrant women from quite diverse regions in the world all faced the same adverse conditions in their country of birth. In that case, one would expect to find variation in regional gender differences, but we did not observe them, at least not for speaking, writing, and listening proficiency in Dutch. Interestingly, for reading proficiency we observed that African female learners and women originating from the Middle East scored higher on the reading test than their male counterparts. When this is claimed to provide evidence for a gender-related acculturation scenario, we cannot explain the absence of regional differences in the other three skills. Although we do not reject gender-related acculturation explanations in general, we want to put forward a plain observation that provides an alternative explanation for this remarkable outcome. If one checks S3 Table, it can be observed that, without exception, the male-female ratio of language learners from Africa and the Middle East is larger than one. This is very uncommon for language learners from other regions. Even for Indonesia, the largest Muslim country in the world, the male-female ratio is smaller than one. In our view, these male-female ratios reflect the degree to which women in Islamic countries have the freedom to leave their homes to communicate with other people than their families and to learn the language of the target country. If opportunities to leave home are limited, the only language skill remaining they can focus on and excel in, is reading. This may be seen of course as a gender-related acculturation pattern, but it precludes gender-related acculturation processes offering an overall explanatory scenario for women outperforming men in second language proficiency, let alone that this approach can cope with the gender distinctions between reading, listening, writing, and speaking.

Turning to more nature-based explanatory schemes, it is obvious that they are commonly put forward and accepted as being valid to explain differences between boys and girls in L1 acquisition. It was also evident that most studies in that field were dealing with productive language skills, perhaps on the assumption that productive skills reflect the most substantial part of language proficiency. On the basis of a large L1 meta-analytic study with more than 4 million students it was concluded that female students fared better in writing and language use (grammatical conventions, expression, spelling, etc.), in productive language skills therefore [6, 9]. Smaller but consistent effect sizes in favor of female students were detected for reading and verbal reasoning. That confirms our findings that the more outspoken differences are found for productive language skills.

The distinction between the four language modalities needs to worked out and tested in greater detail on the cognitive and language resources they require, both in L1 and L2 acquisition. One important approach would be to figure out which specific speaking skill components contribute to the observed gender gap. Is it vocabulary, pronunciation, word and sentence formation (morphology and syntax), a combination of these components, or is it communicative competence? The speaking proficiency test used in the present study does not differentiate between these components. Earlier research has found that women are more concerned about pronunciation accuracy [78] and conform more to the standard form of the L2 [79]. Future research might profit from more differentiated speaking test scores.

Assuming a nature-based, genetic difference in the female and male equipment in L1 and L2 acquisition does not preclude that nature (genes) and nurture (environment) interact in intricate ways and on various levels ranging from the individual to the societal [29–33]. The research outcomes we provided give strong circumstantial evidence of an initially nature-based gender distinction. The gender gap in favor of L2 female learners in speaking and writing turned out to be a robust, convincing effect that requires further research, both in other language combinations and for lower educational levels. We also need additional fundamental research on the underlying cognitive and language processes in male and female brains.

## Supporting Information

S1 Fig Density Plots of the Distribution of the Raw Scores of Speaking Proficiency in Dutch against Years of Secondary Education (left panel) and the Distribution of the Residuals of Speaking Proficiency of the Final Model without the Effects of Gender, Education, and their Interaction against Years of Secondary Education (centered), Combined with the Regression Slopes of Education for Male and Female Learners, Extracted from the Final Model in Table 2 (right panel). (TIF)

S2 Fig Density Plots of the Distribution of the Raw Scores of Writing Proficiency in Dutch against Years of Secondary Education (left panel) and the Distribution of the Residuals of Writing Proficiency of the Final Model without the Effects of Gender, Education, and their Interaction against Years of Secondary Education (centered), Combined with the Regression Slopes of Education for Male and Female Learners, Extracted from the Final Model in Table 3 (right panel). (TIF)

S3 Fig Density Plots of the Distribution of the Raw Scores of Reading Proficiency in Dutch against Years of Secondary Education (left panel) and the Distribution of the Residuals of Reading Proficiency of the Final Model without the Effects of Gender, Education, and their Interaction against Years of Secondary Education (centered), Combined with the Regression Slopes of Education for Male and Female Learners, Extracted from the Final Model in Table 4 (right panel). (TIF)

S4 Fig Density Plots of the Distribution of the Raw Scores of Listening Proficiency in Dutch against Years of Secondary Education (left panel) and the Distribution of the Residuals of Listening Proficiency of the Final Model without the Effects of Gender, Education, and their Interaction against Years of Secondary Education (centered), Combined with the Regression Slopes of Education for Male and Female Learners, Extracted from the Final Model in Table 5 (right panel). (TIF)

S1 Table Mean Speaking Scores (SD) of Male and Female learners, T-tests and P-values. (DOCX)

S2 Table Mean Writing Scores (SD) of Male and Female Learners, T-tests and P-values. (DOCX)

S3 Table Mean Reading Scores (SD) of Male and Female Learners, T-tests and P-values. (DOCX)

S4 Table Mean Listening Scores (SD) of Male and Female Learners, T-tests and P-values. (DOCX)
