# Supplementary figures and images for: The Gender Gap in Second Language Acquisition: Gender Differences in the Acquisition of Dutch among Immigrants from 88 Countries with 49 Mother Tongues
Source: PLoS One. 2015 Nov 5;10(11):e0142056. doi: 10.1371/journal.pone.0142056 (PMC4634989; doi:10.1371/journal.pone.0142056)

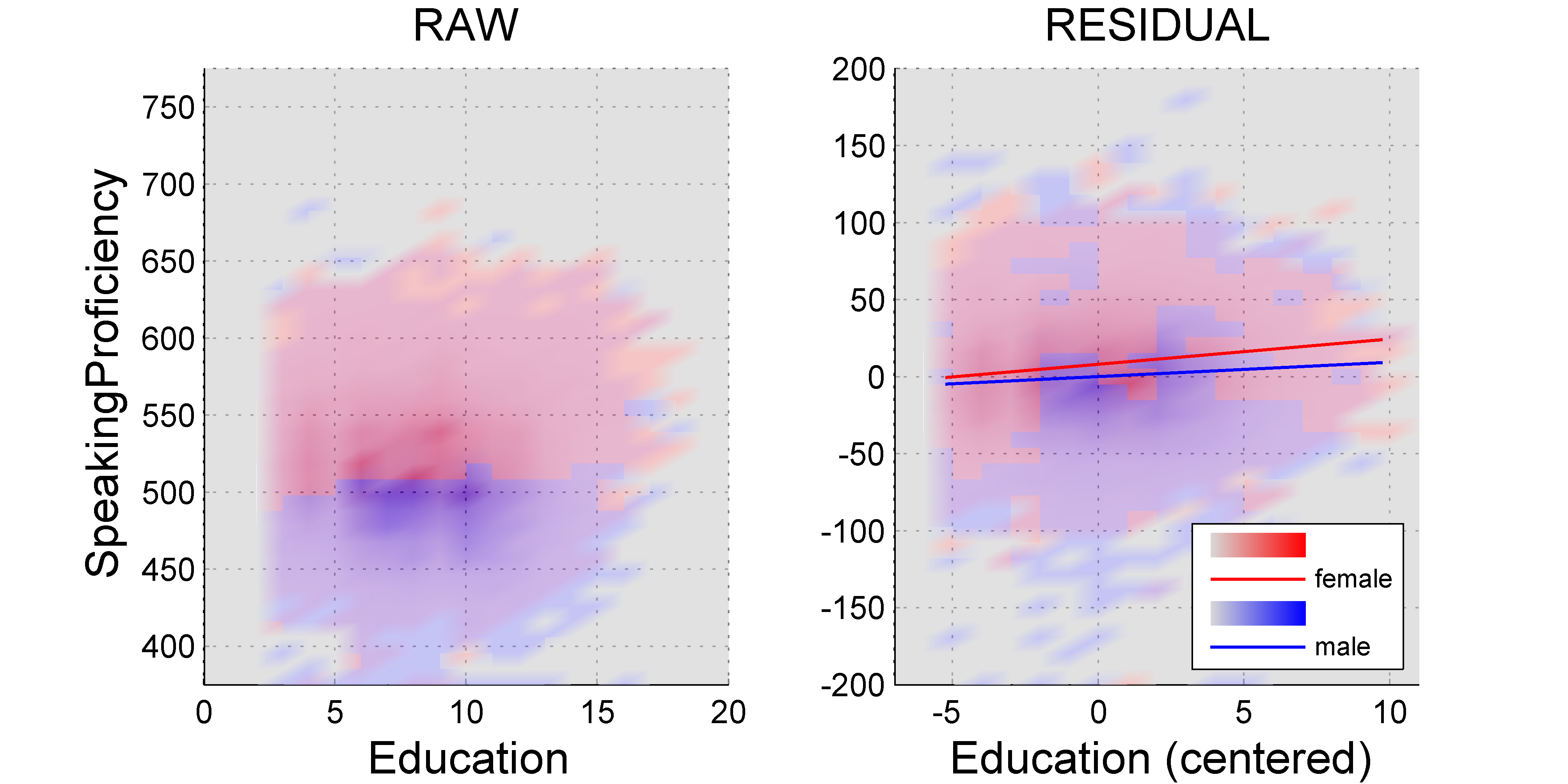

Supplement: S1 Fig — (TIF) [file pone.0142056.s001.tif]

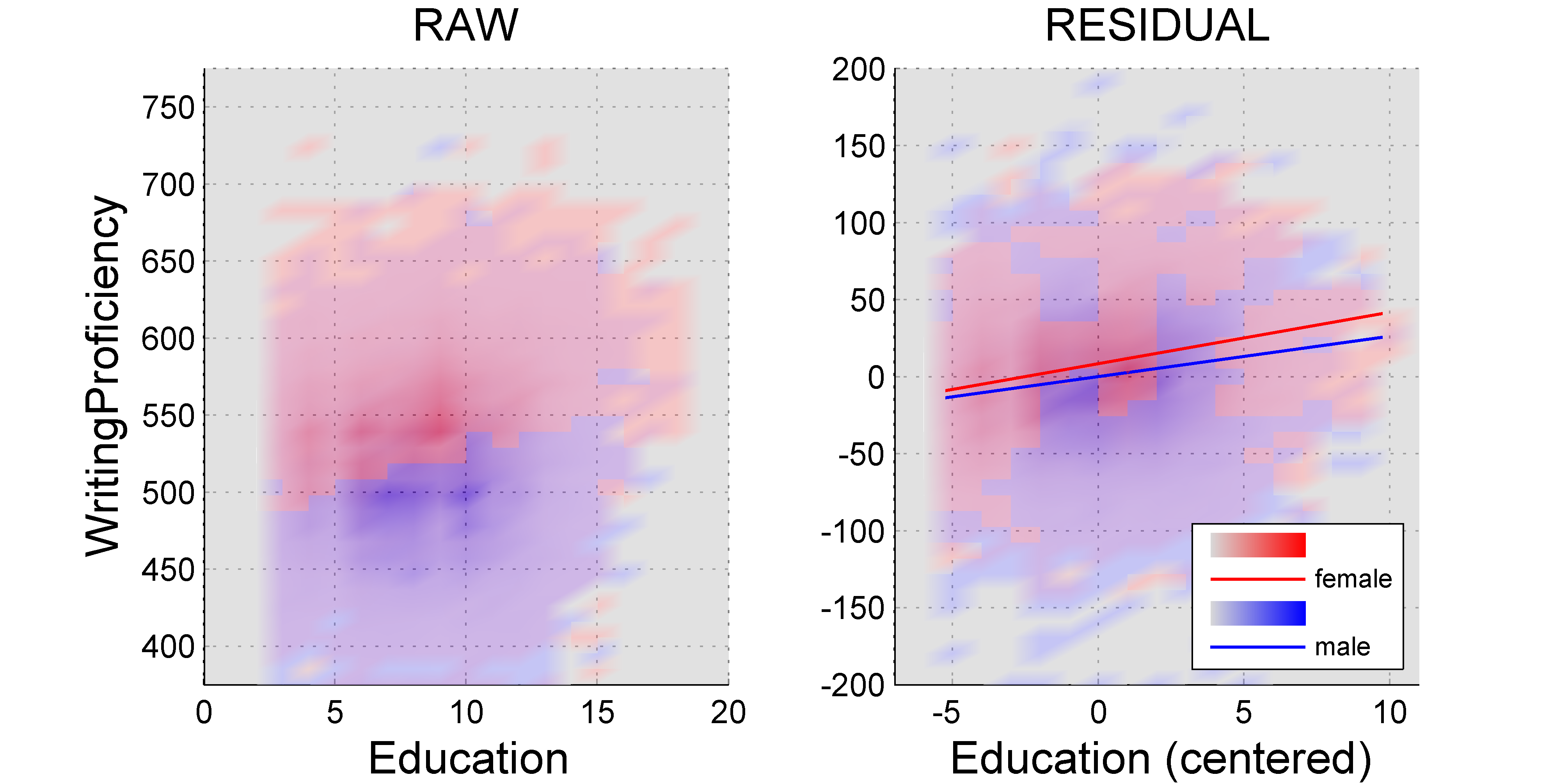

Supplement: S2 Fig — (TIF) [file pone.0142056.s002.tif]

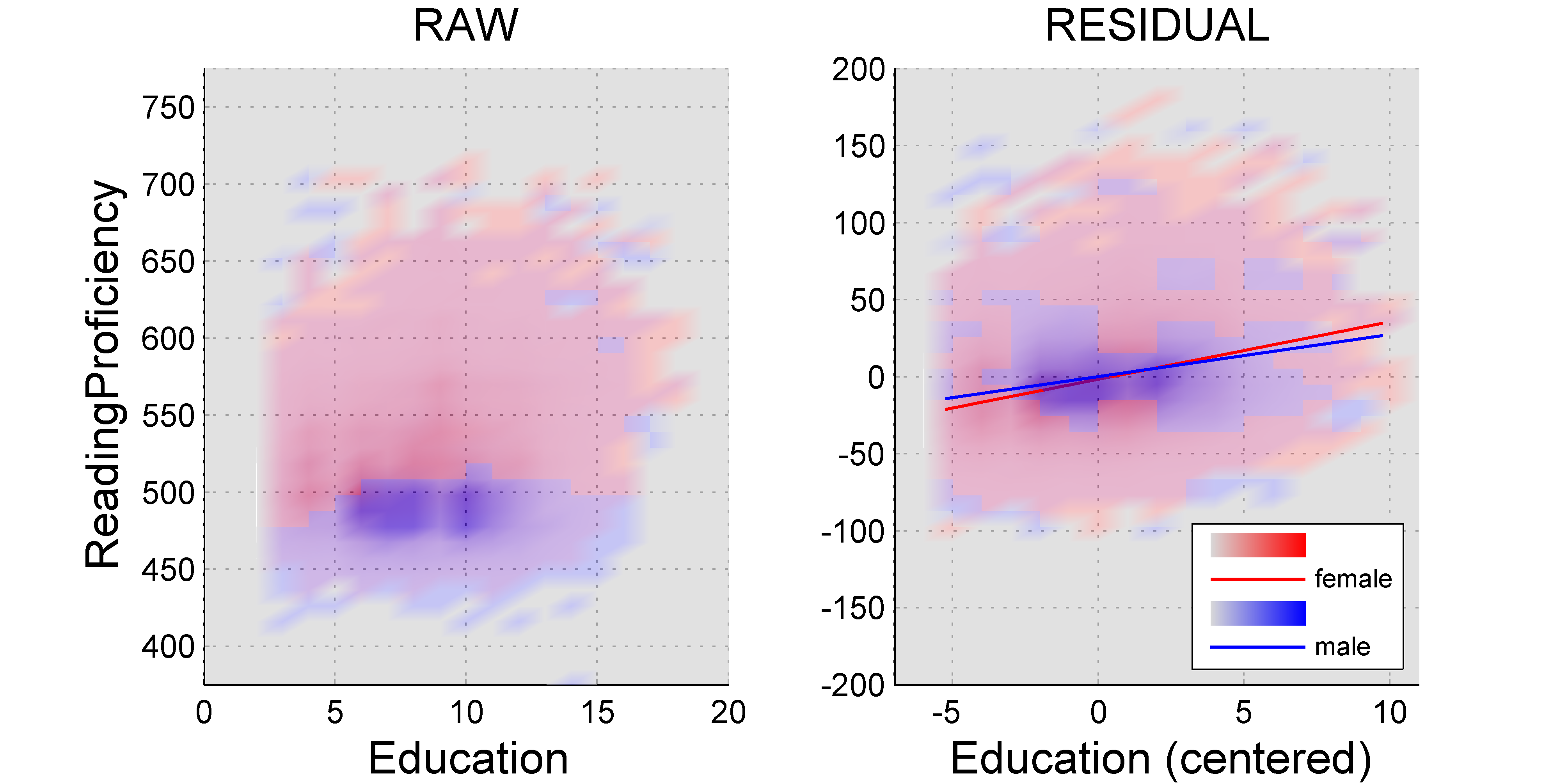

Supplement: S3 Fig — (TIF) [file pone.0142056.s003.tif]

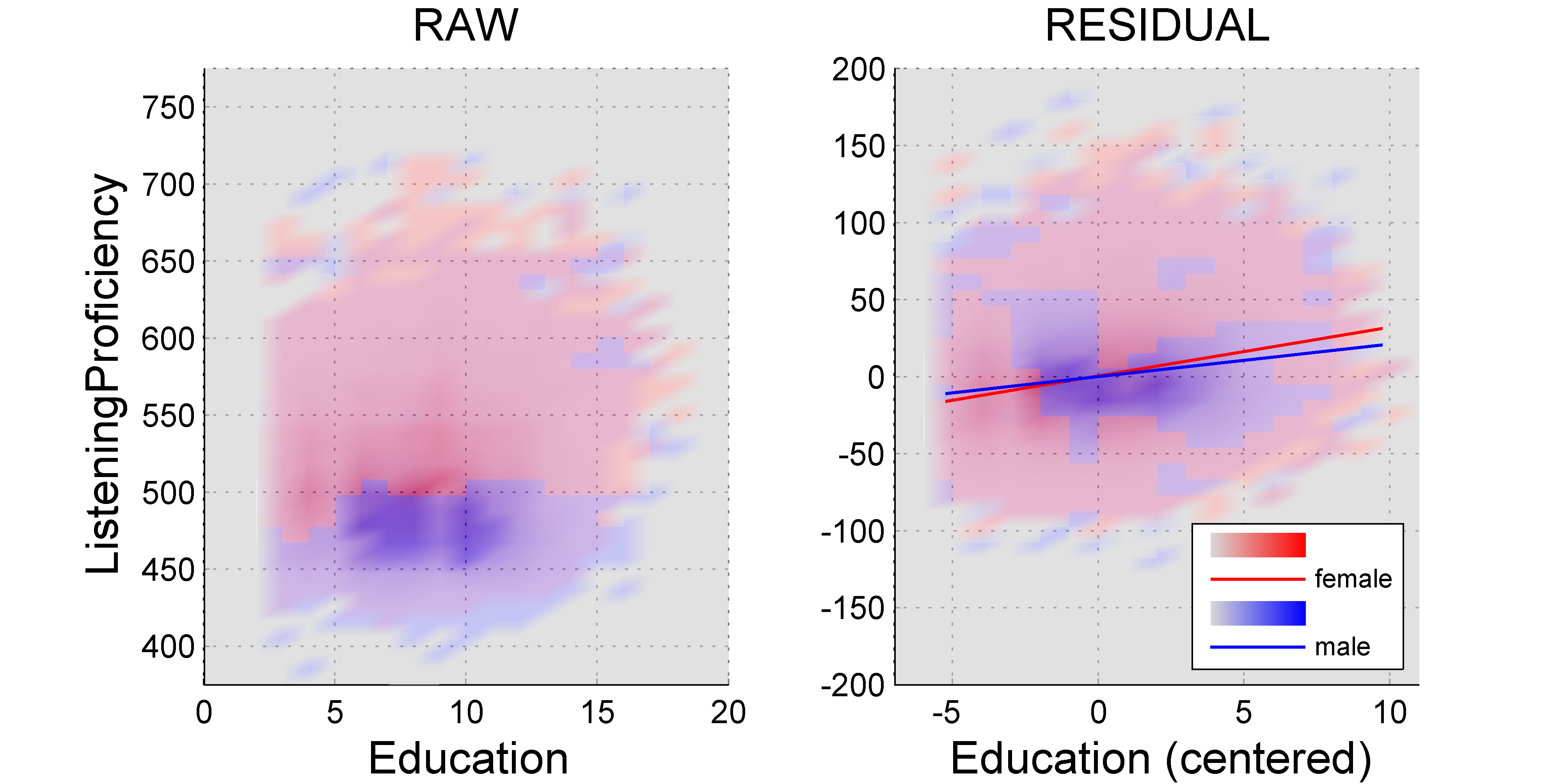

Supplement: S4 Fig — (TIF) [file pone.0142056.s004.tif]
