# Supplementary material for: The Gender Gap in Second Language Acquisition: Gender Differences in the Acquisition of Dutch among Immigrants from 88 Countries with 49 Mother Tongues
Source: PLoS One. 2015 Nov 5;10(11):e0142056. doi: 10.1371/journal.pone.0142056 (PMC4634989; doi:10.1371/journal.pone.0142056)
Supplement: S1 Table — (DOCX) [file pone.0142056.s005.docx]

| Country | Males Mean (SD) | n | Females Mean (SD) | n | T–test | p–value |
| --- | --- | --- | --- | --- | --- | --- |
| Afghanistan | 493 (29) | 468 | 497 (29) | 235 | 1.96 | .051 |
| Albania | 514 (35) | 25 | 515 (34) | 41 | .19 | .852 |
| Algeria | 495 (30) | 103 | 509 (32) | 38 | 2.40 | .018 |
| Angola | 487 (38) | 15 | 497 (32) | 17 | .85 | .405 |
| Argentina | 525 (36) | 24 | 524 (25) | 93 | –.23 | .822 |
| Armenia | 500 (33) | 74 | 510 (30) | 145 | 2.16 | .027 |
| Australia | 535 (32) | 26 | 534 (31) | 83 | –.13 | .895 |
| Austria | 561 (30) | 18 | 568 (30) | 141 | .84 | .400 |
| Belgium | 528 (29) | 23 | 528 (26) | 39 | .02 | .982 |
| Bolivia | 508 (29) | 6 | 507 (25) | 24 | –.10 | .924 |
| Brazil | 514 (31) | 87 | 513 (33) | 310 | –.37 | .711 |
| Bulgaria | 528 (34) | 30 | 531 (33) | 257 | .46 | .649 |
| Burundi | 488 (28) | 44 | 494 (32) | 29 | .78 | .441 |
| Cameroon | 490 (35) | 36 | 484 (34) | 21 | –.60 | .553 |
| Canada | 543 (33) | 20 | 539 (37) | 96 | –.39 | .697 |
| Cape Verde | 510 (19) | 15 | 504 (23) | 18 | –.76 | .454 |
| Chile | 498 (35) | 13 | 509 (31) | 57 | 1.20 | .233 |
| China | 471 (42) | 72 | 491 (34) | 256 | 4.30 | <.001 |
| Colombia | 509 (34) | 55 | 507 (30) | 187 | –.49 | .625 |
| Congo, Dem. Rep. | 479 (29) | 31 | 505 (33) | 8 | 2.24 | .031 |
| Congo, Rep. | 493 (26) | 27 | 508 (30) | 9 | 1.45 | .158 |
| Costa Rica | 520 (18) | 3 | 518 (19) | 16 | –.16 | .827 |
| Croatia | 506 (34) | 129 | 519 (29) | 220 | 3.56 | <.001 |
| Cuba | 510 (26) | 22 | 507 (28) | 34 | –.41 | .685 |
| Czech Rep. | 548 (39) | 18 | 541 (32) | 294 | –.83 | .409 |
| Denmark | 544 (24) | 15 | 552 (33) | 107 | .82 | .413 |
| Dominican Rep. | 484 (7) | 4 | 504 (35) | 28 | 1.13 | .269 |
| Ecuador | 514 (34) | 13 | 510 (31) | 41 | –.43 | .668 |
| Egypt | 498 (38) | 152 | 507 (38) | 52 | 1.45 | .150 |
| Eritrea | 489 (35) | 15 | 475 (42) | 9 | –.88 | .391 |
| Estonia | 556 (0) | 1 | 546 (27) | 30 | –.35 | .726 |
| Ethiopia | 482 (29) | 60 | 492 (38) | 18 | 1.14 | .257 |
| Finland | 531 (33) | 16 | 539 (32) | 146 | .95 | .345 |
| France | 529 (34) | 104 | 529 (35) | 473 | .04 | .971 |
| Georgia | 506 (27) | 12 | 499 (31) | 27 | –.68 | .502 |
| Germany | 557 (35) | 351 | 564 (36) | 1546 | 3.24 | .001 |
| Greece | 518 (35) | 68 | 524 (35) | 75 | .93 | .352 |
| Guatemala | 502 (23) | 6 | 524 (39) | 16 | 1.27 | .220 |
| Hong Kong | 498 (63) | 8 | 489 (37) | 39 | –.53 | .577 |
| Hungary | 535 (37) | 32 | 538 (30) | 378 | .54 | .594 |
| Iceland | 532 (34) | 10 | 544 (36) | 19 | .92 | .362 |
| India | 502 (33) | 21 | 518 (31) | 53 | 1.98 | .052 |
| Indonesia | 487 (28) | 210 | 500 (30) | 679 | 5.42 | <.001 |
| Iran | 495 (31) | 752 | 501 (29) | 650 | 4.05 | <.001 |

| Country | Males Mean (SD) | N | Females Mean (SD) | N | T–test | p–value |
| --- | --- | --- | --- | --- | --- | --- |
| Iraq | 491 (29) | 907 | 496 (30) | 363 | 2.96 | .003 |
| Ireland | 527 (24) | 20 | 541 (33) | 85 | 2.15 | .038 |
| Italy | 527 (38) | 131 | 529 (34) | 248 | .52 | .591 |
| Japan | 478 (31) | 11 | 497 (32) | 163 | 1.89 | .068 |
| Jordan | 504 (28) | 15 | 503 (25) | 9 | –.04 | .971 |
| Korea Rep. | 475 (14) | 3 | 497 (26) | 31 | 1.43 | .164 |
| Kuwait | 500 (31) | 15 | 519 (33) | 12 | 1.52 | .142 |
| Latvia | 479 (0) | 1 | 529 (28) | 27 | 1.75 | .092 |
| Lebanon | 495 (48) | 28 | 513 (39) | 20 | 1.34 | .188 |
| Liberia | 477 (35) | 18 | 492 (39) | 4 | .75 | .461 |
| Lithuania | 522 (24) | 4 | 526 (30) | 78 | .24 | .813 |
| Malaysia | 503 (44) | 9 | 503 (25) | 44 | .04 | .979 |
| Mexico | 498 (24) | 22 | 508 (31) | 146 | 1.50 | .135 |
| Morocco | 489 (35) | 1526 | 505 (35) | 697 | 10.18 | <.001 |
| Netherlands | 517 (44) | 145 | 532 (46) | 132 | 2.79 | .006 |
| New Zealand | 533 (45) | 10 | 539 (34) | 28 | .41 | .687 |
| Nigeria | 492 (36) | 55 | 487 (41) | 21 | –.57 | .568 |
| Norway | 548 (32) | 18 | 556 (35) | 90 | .87 | .385 |
| Peru | 503 (42) | 31 | 510 (28) | 151 | .86 | .395 |
| Philippines | 500 (24) | 14 | 498 (27) | 183 | –.33 | .743 |
| Poland | 526 (28) | 74 | 527 (30) | 1374 | .31 | .760 |
| Portugal | 513 (36) | 19 | 524 (30) | 88 | 1.39 | .169 |
| Romania | 524 (33) | 65 | 526 (31) | 444 | .42 | .675 |
| Russian Federation | 518 (36) | 212 | 521 (30) | 1367 | 1.07 | .287 |
| Rwanda | 483 (24) | 63 | 491 (27) | 52 | 1.55 | .124 |
| Serbia | 506 (30) | 658 | 516 (30) | 1011 | 6.37 | <.001 |
| Singapore | 533 (53) | 3 | 544 (34) | 25 | .48 | .634 |
| Somalia | 477 (31) | 206 | 481 (30) | 44 | .87 | .387 |
| South Africa | 533 (33) | 47 | 541 (31) | 92 | 1.49 | .138 |
| Spain | 516 (36) | 87 | 520 (31) | 445 | 1.14 | .256 |
| Sri Lanka | 478 (25) | 31 | 478 (25) | 24 | .03 | .977 |
| Sudan | 481 (28) | 199 | 489 (26) | 46 | 1.73 | .085 |
| Sweden | 543 (27) | 24 | 557 (36) | 144 | 1.87 | .064 |
| Switzerland | 557 (31) | 34 | 575 (37) | 107 | 2.47 | .015 |
| Syria | 498 (35) | 104 | 510 (38) | 50 | 1.96 | .052 |
| Thailand | 486 (34) | 15 | 490 (30) | 115 | .57 | .572 |
| Tunisia | 505 (35) | 55 | 508 (37) | 29 | .33 | .745 |
| Turkey | 486 (40) | 911 | 497 (38) | 564 | 5.22 | <.001 |
| Ukraine | 517 (36) | 9 | 523 (28) | 112 | .57 | .569 |
| United Kingdom | 532 (35) | 212 | 538 (31) | 423 | 2.467 | .014 |
| United States | 528 (31) | 134 | 535 (35) | 334 | 1.76 | .078 |
| Uruguay | 537 (33) | 5 | 542 (42) | 20 | .24 | .811 |
| Venezuela | 522 (28) | 15 | 506 (31) | 86 | –1.93 | .057 |
| Vietnam | 478 (30) | 26 | 489 (29) | 77 | 1.60 | .105 |

**S1 Appendix 1.**Mean speaking scores (SD) of male and female learners, T-tests and p-values.
