# Supplementary material for: The Gender Gap in Second Language Acquisition: Gender Differences in the Acquisition of Dutch among Immigrants from 88 Countries with 49 Mother Tongues
Source: PLoS One. 2015 Nov 5;10(11):e0142056. doi: 10.1371/journal.pone.0142056 (PMC4634989; doi:10.1371/journal.pone.0142056)
Supplement: S2 Table — (DOCX) [file pone.0142056.s006.docx]

| Country | Males Mean (SD) | n | Females Mean (SD) | N | T-test | p-value |
| --- | --- | --- | --- | --- | --- | --- |
| Afghanistan | 498 (42) | 469 | 494 (40) | 237 | –1.41 | .150 |
| Albania | 517 (36) | 24 | 521 (43) | 41 | .41 | .686 |
| Algeria | 497 (39) | 103 | 525 (47) | 37 | 3.55 | .001 |
| Angola | 488 (49) | 15 | 514 (47) | 17 | 1.56 | .129 |
| Argentina | 530 (39) | 24 | 538 (32) | 95 | 1.13 | .263 |
| Armenia | 504 (37) | 76 | 518 (38) | 145 | 2.39 | .018 |
| Australia | 543 (27) | 27 | 549 (42) | 83 | .68 | .500 |
| Austria | 570 (46) | 18 | 586 (35) | 142 | 1.69 | .092 |
| Belgium | 543 (40) | 22 | 544 (36) | 40 | .13 | .898 |
| Bolivia | 525 (41) | 6 | 519 (45) | 24 | –.28 | .778 |
| Brazil | 520 (44) | 87 | 526 (42) | 316 | 1.21 | .227 |
| Bulgaria | 532 (37) | 30 | 547 (44) | 257 | 1.76 | .808 |
| Burundi | 517 (34) | 46 | 515 (35) | 29 | –.22 | .824 |
| Cameroon | 517 (33) | 36 | 493 (46) | 21 | –2.06 | .048 |
| Canada | 547 (35) | 21 | 556 (40) | 95 | 1.06 | .333 |
| Cape Verde | 506 (35) | 16 | 494 (35) | 18 | –.99 | .328 |
| Chile | 487 (53) | 12 | 524 (35) | 58 | 3.06 | .003 |
| China | 511 (42) | 74 | 523 (38) | 267 | 2.42 | .016 |
| Colombia | 518 (47) | 55 | 516 (42) | 187 | –.27 | .788 |
| Congo, Dem. Rep. | 492 (37) | 32 | 509 (30) | 8 | 1.16 | .255 |
| Congo, Rep. | 498 (43) | 27 | 501 (46) | 9 | .22 | .828 |
| Costa Rica | 517 (12) | 3 | 530 (29) | 16 | .75 | .466 |
| Croatia | 514 (40) | 135 | 523 (37) | 223 | 2.10 | .036 |
| Cuba | 532 (49) | 22 | 519 (37) | 34 | –1.22 | .230 |
| Czech Rep. | 572 (40) | 18 | 555 (39) | 297 | –1.81 | .071 |
| Denmark | 549 (27) | 16 | 564 (40) | 105 | 1.44 | .152 |
| Dominican Rep. | 491 (36) | 4 | 508 (33) | 29 | .98 | .333 |
| Ecuador | 516 (48) | 15 | 522 (45) | 40 | .43 | .673 |
| Egypt | 490 (51) | 155 | 508 (44) | 52 | 2.28 | .024 |
| Eritrea | 497 (41) | 15 | 480 (55) | 9 | –.88 | .391 |
| Estonia | 595 (0) | 1 | 577 (43) | 30 | –.42 | .677 |
| Ethiopia | 490 (41) | 61 | 483 (38) | 18 | –.65 | .519 |
| Finland | 552 (36) | 15 | 566 (39) | 149 | 1.28 | .203 |
| France | 545 (42) | 104 | 550 (39) | 473 | 1.20 | .232 |
| Georgia | 504 (27) | 11 | 508 (40) | 27 | .36 | .720 |
| Germany | 571 (42) | 350 | 580 (39) | 1548 | 3.53 | <.001 |
| Greece | 523 (43) | 69 | 539 (35) | 76 | 2.43 | .016 |
| Guatemala | 522 (36) | 6 | 528 (40) | 16 | .31 | .761 |
| Hong Kong | 536 (55) | 8 | 509 (44) | 38 | –1.54 | .131 |
| Hungary | 551 (36) | 32 | 553 (40) | 387 | .28 | .783 |
| Iceland | 555 (45) | 10 | 553 (39) | 19 | –.204 | .840 |
| India | 513 (36) | 22 | 532 (36) | 54 | 2.01 | .048 |
| Indonesia | 495 (43) | 210 | 512 (37) | 681 | 5.53 | < .001 |
| Iran | 493 (40) | 783 | 498 (38) | 665 | 2.51 | .012 |

| Country | Males Mean (SD) | N | Females Mean (SD) | N | T-test | p-value |
| --- | --- | --- | --- | --- | --- | --- |
| Iraq | 494 (39) | 913 | 503 (38) | 365 | 3.79 | <.001 |
| Ireland | 534 (38) | 20 | 552 (40) | 87 | 1.80 | .074 |
| Italy | 533 (51) | 130 | 547 (44) | 250 | 2.90 | .004 |
| Japan | 497 (47) | 10 | 528 (41) | 163 | 2.35 | .020 |
| Jordan | 506 (38) | 16 | 506 (49) | 9 | –.03 | .976 |
| Korea Rep. | 503 (29) | 3 | 516 (32) | 31 | .66 | .514 |
| Kuwait | 521 (50) | 13 | 515 (52) | 12 | –.31 | .762 |
| Latvia | 483 (0) | 1 | 549 (45) | 27 | 1.43 | .166 |
| Lebanon | 500 (46) | 28 | 516 (40) | 21 | 1.26 | .215 |
| Liberia | 470 (59) | 17 | 505 (46) | 4 | 1.10 | .283 |
| Lithuania | 545 (43) | 4 | 539 (33) | 78 | –.33 | .740 |
| Malaysia | 527 (40) | 9 | 520 (39) | 49 | –.51 | .613 |
| Mexico | 502 (41) | 22 | 524 (38) | 149 | 2.51 | .013 |
| Morocco | 495 (42) | 1557 | 509 (41) | 719 | 7.48 | <.001 |
| Netherlands | 518 (47) | 143 | 527 (42) | 134 | 1.61 | .108 |
| New Zealand | 549 (53) | 10 | 556 (21) | 30 | .47 | .639 |
| Nigeria | 499 (40) | 56 | 500 (39) | 23 | .16 | .873 |
| Norway | 544 (43) | 20 | 564 (41) | 93 | 2.04 | .044 |
| Peru | 513 (43) | 31 | 526 (39) | 152 | 1.76 | .081 |
| Philippines | 501 (45) | 14 | 502 (35) | 184 | .04 | .968 |
| Poland | 536 (36) | 74 | 537 (40) | 1389 | .28 | .778 |
| Portugal | 524 (43) | 20 | 534 (45) | 90 | .93 | .353 |
| Romania | 526 (44) | 66 | 543 (40) | 448 | 3.18 | .002 |
| Russian Federation | 533 (47) | 215 | 542 (41) | 1371 | 2.69 | .008 |
| Rwanda | 515 (34) | 66 | 520 (37) | 51 | .75 | .454 |
| Serbia | 515 (37) | 660 | 523 (38) | 1020 | 4.38 | <.001 |
| Singapore | 554 (65) | 3 | 550 (46) | 25 | –.17 | .870 |
| Somalia | 484 (42) | 212 | 483 (46) | 43 | –.15 | .883 |
| South Africa | 537 (45) | 47 | 546 (38) | 92 | 1.30 | .197 |
| Spain | 535 (44) | 90 | 543 (39) | 455 | 1.72 | .085 |
| Sri Lanka | 490 (31) | 31 | 484 (36) | 24 | –.63 | .531 |
| Sudan | 493 (41) | 198 | 491 (43) | 47 | .22 | .826 |
| Sweden | 565 (45) | 25 | 570 (39) | 144 | .58 | .563 |
| Switzerland | 574 (38) | 34 | 590 (36) | 107 | 2.09 | .039 |
| Syria | 502 (43) | 107 | 505 (40) | 52 | .49 | .624 |
| Thailand | 504 (36) | 15 | 503 (43) | 115 | –.04 | .970 |
| Tunisia | 499 (38) | 58 | 512 (46) | 29 | 1.34 | .184 |
| Turkey | 487 (46) | 933 | 501 (48) | 570 | 5.68 | <.001 |
| Ukraine | 538 (45) | 9 | 537 (40) | 111 | –.04 | 969 |
| United Kingdom | 539 (39) | 213 | 550 (41) | 428 | 3.11 | .002 |
| United States | 539 (39) | 137 | 549 (42) | 334 | 2.40 | .017 |
| Uruguay | 561 (41) | 5 | 539 (47) | 20 | –.96 | .349 |
| Venezuela | 501 (54) | 15 | 514 (39) | 86 | 1.14 | .256 |
| Vietnam | 506 (47) | 27 | 509 (39) | 78 | .40 | .691 |

**S2 Appendix 2.** Mean writing scores (SD) of male and female learners, T-tests and p-values.
