# Supplementary material for: The Gender Gap in Second Language Acquisition: Gender Differences in the Acquisition of Dutch among Immigrants from 88 Countries with 49 Mother Tongues
Source: PLoS One. 2015 Nov 5;10(11):e0142056. doi: 10.1371/journal.pone.0142056 (PMC4634989; doi:10.1371/journal.pone.0142056)
Supplement: S3 Table — (DOCX) [file pone.0142056.s007.docx]

| Country | Males Mean (SD) | n | Females Mean (SD) | N | T-test | p-value |
| --- | --- | --- | --- | --- | --- | --- |
| Afghanistan | 487 (21) | 476 | 485 (22) | 238 | –1.65 | .100 |
| Albania | 514 (25) | 25 | 513 (31) | 41 | –.10 | .922 |
| Algeria | 492 (20) | 106 | 505 (22) | 38 | 3.18 | .002 |
| Angola | 483 (23) | 15 | 510 (39) | 17 | 1.54 | .135 |
| Argentina | 523 (24) | 24 | 521 (28) | 94 | –.37 | .715 |
| Armenia | 498 (26) | 79 | 501 (25) | 147 | .92 | .361 |
| Australia | 555 (42) | 27 | 553 (38) | 83 | –.25 | .800 |
| Austria | 582 (39) | 17 | 567 (37) | 142 | –1.54 | .127 |
| Belgium | 544 (43) | 23 | 533 (30) | 41 | –1.24 | .221 |
| Bolivia | 492 (34) | 6 | 509 (33) | 25 | 1.13 | .267 |
| Brazil | 519 (32) | 88 | 517 (34) | 323 | –.61 | .541 |
| Bulgaria | 534 (44) | 31 | 529 (34) | 258 | –.80 | .423 |
| Burundi | 496 (21) | 46 | 497 (25) | 29 | .15 | .877 |
| Cameroon | 501 (29) | 36 | 487 (26) | 21 | –1.78 | .081 |
| Canada | 561 (44) | 22 | 555 (35) | 98 | –.58 | .568 |
| Cape Verde | 496 (21) | 16 | 490 (18) | 19 | –1.01 | .319 |
| Chile | 502 (24) | 13 | 514 (32) | 59 | 1.20 | .236 |
| China | 509 (29) | 76 | 508 (27) | 272 | –.12 | .903 |
| Colombia | 517 (32) | 56 | 505 (26) | 194 | –2.60 | .011 |
| Congo, Dem. Rep. | 488 (15) | 31 | 490 (18) | 8 | .34 | .739 |
| Congo, Rep. | 494 (22) | 27 | 503 (29) | 9 | 1.06 | .296 |
| Costa Rica | 526 (7) | 3 | 504 (29) | 17 | –1.30 | .209 |
| Croatia | 527 (34) | 139 | 521 (27) | 227 | –1.69 | .092 |
| Cuba | 513 (30) | 22 | 512 (7) | 34 | –.12 | .904 |
| Czech Rep. | 556 (34) | 18 | 539 (35) | 298 | –1.88 | .061 |
| Denmark | 564 (36) | 17 | 561 (35) | 107 | –.21 | .836 |
| Dominican Rep. | 485 (22) | 4 | 492 (32) | 28 | .41 | .683 |
| Ecuador | 508 (26) | 15 | 504 (24) | 42 | –.44 | .663 |
| Egypt | 493 (24) | 163 | 493 (24) | 53 | .25 | .804 |
| Eritrea | 493 (22) | 16 | 489 (21) | 10 | –.50 | .621 |
| Estonia | 560 (0) | 1 | 549 (36) | 30 | –.32 | .755 |
| Ethiopia | 498 (22) | 63 | 493 (31) | 18 | –.76 | .448 |
| Finland | 578 (47) | 16 | 563 (40) | 152 | –1.40 | .165 |
| France | 542 (38) | 102 | 534 (35) | 486 | –2.08 | .038 |
| Georgia | 509 (21) | 12 | 500 (23) | 27 | –1.12 | .272 |
| Germany | 574 (44) | 354 | 566 (41) | 1562 | –2.96 | .003 |
| Greece | 529 (36) | 69 | 528 (39) | 78 | –.09 | .931 |
| Guatemala | 510 (23) | 6 | 506 (24) | 17 | –.36 | .721 |
| Hong Kong | 517 (42) | 8 | 503 (25) | 41 | –.92 | .385 |
| Hungary | 542 (42) | 33 | 541 (38) | 393 | –.14 | .887 |
| Iceland | 553 (23) | 10 | 540 (31) | 19 | –1.12 | .275 |
| India | 515 (23) | 23 | 518 (33) | 55 | .41 | .682 |
| Indonesia | 503 (28) | 215 | 507 (27) | 703 | 1.75 | .081 |
| Iran | 493 (25) | 790 | 491 (22) | 680 | –1.89 | .058 |

| Country | Males Mean (SD) | N | Females Mean (SD) | N | T-test | p-value |
| --- | --- | --- | --- | --- | --- | --- |
| Iraq | 490 (22) | 922 | 490 (20) | 377 | .14 | .887 |
| Ireland | 539 (35) | 22 | 542 (34) | 87 | .3 | .712 |
| Italy | 535 (35) | 131 | 534 (38) | 259 | –.13 | .897 |
| Japan | 510 (20) | 11 | 511 (29) | 172 | .17 | .863 |
| Jordan | 488 (23) | 16 | 500 (21) | 9 | 1.27 | .216 |
| Korea Rep. | 509 (15) | 3 | 513 (25) | 32 | .25 | .804 |
| Kuwait | 505 (24) | 14 | 502 (29) | 12 | –.27 | .788 |
| Latvia | 509 (0) | 1 | 538 (43) | 27 | .67 | .507 |
| Lebanon | 506 (37) | 28 | 505 (26) | 21 | –.13 | .899 |
| Liberia | 494 (18) | 17 | 487 (23) | 4 | –.65 | .523 |
| Lithuania | 542 (29) | 4 | 526 (30) | 80 | –1.07 | .288 |
| Malaysia | 528 (36) | 9 | 509 (24) | 51 | –1.55 | .155 |
| Mexico | 498 (25) | 21 | 516 (31) | 148 | 2.59 | .010 |
| Morocco | 487 (21) | 1597 | 492 (22) | 721 | 4.45 | < .001 |
| Netherlands | 504 (31) | 147 | 508 (31) | 134 | 1.24 | .216 |
| New Zealand | 553 (32) | 11 | 557 (45) | 30 | .28 | .780 |
| Nigeria | 505 (25) | 58 | 496 (27) | 22 | –1.41 | .164 |
| Norway | 564 (35) | 20 | 553 (37) | 93 | –1.12 | .265 |
| Peru | 509 (30) | 31 | 511 (30) | 157 | .33 | .740 |
| Philippines | 508 (25) | 14 | 495 (23) | 190 | –1.92 | .056 |
| Poland | 536 (39) | 74 | 526 (35) | 1428 | –2.57 | .010 |
| Portugal | 525 (33) | 20 | 527 (32) | 94 | .32 | .753 |
| Romania | 536 (42) | 167 | 529 (34) | 452 | –1.28 | .203 |
| Russian Federation | 534 (43) | 219 | 526 (34) | 1393 | –2.88 | .004 |
| Rwanda | 494 (22) | 66 | 496 (18) | 53 | .50 | .615 |
| Serbia | 522 (29) | 683 | 519 (32) | 1047 | –2.12 | .034 |
| Singapore | 532 (41) | 3 | 554 (57) | 25 | .65 | .523 |
| Somalia | 489 (21) | 217 | 484 (20) | 43 | –1.28 | .202 |
| South Africa | 550 (37) | 48 | 546 (34) | 94 | –.61 | .546 |
| Spain | 532 (35) | 88 | 531 (35) | 457 | –.12 | .908 |
| Sri Lanka | 495 (14) | 32 | 488 (16) | 25 | –1.71 | .093 |
| Sudan | 490 (24) | 202 | 489 (24) | 47 | –.14 | .887 |
| Sweden | 570 (36) | 25 | 580 (43) | 146 | .123 | .262 |
| Switzerland | 583 (43) | 34 | 580 (42) | 108 | –.34 | .736 |
| Syria | 494 (23) | 109 | 493 (21) | 51 | –.32 | .747 |
| Thailand | 492 (22) | 15 | 497 (24) | 118 | .84 | .402 |
| Tunisia | 494 (21) | 56 | 496 (25) | 29 | .53 | .598 |
| Turkey | 493 (24) | 952 | 497 (30) | 577 | 3.03 | .003 |
| Ukraine | 536 (38) | 9 | 516 (26) | 112 | –2.02 | .045 |
| United Kingdom | 551 (37) | 217 | 548 (40) | 433 | –1.04 | .297 |
| United States | 548 (38) | 137 | 553 (40) | 339 | 1.23 | .219 |
| Uruguay | 539 (37) | 5 | 534 (33) | 20 | –.34 | .739 |
| Venezuela | 511 (38) | 16 | 507 (26) | 89 | –.46 | .654 |
| Vietnam | 504 (24) | 25 | 493 (18) | 78 | –2.46 | .016 |

**S3 Appendix 3.** Mean reading scores (SD) of male and female learners, T-tests and p-values.
