# Supplementary material for: The Gender Gap in Second Language Acquisition: Gender Differences in the Acquisition of Dutch among Immigrants from 88 Countries with 49 Mother Tongues
Source: PLoS One. 2015 Nov 5;10(11):e0142056. doi: 10.1371/journal.pone.0142056 (PMC4634989; doi:10.1371/journal.pone.0142056)
Supplement: S4 Table — (DOCX) [file pone.0142056.s008.docx]

| Country | Males Mean (SD) | n | Females Mean (SD) | n | T-test | p-value |
| --- | --- | --- | --- | --- | --- | --- |
| Afghanistan | 482 (24) | 473 | 478 (24) | 236 | –2.11 | .035 |
| Albania | 504 (28) | 25 | 503 (32) | 42 | –.08 | .939 |
| Algeria | 484 (26) | 104 | 495 (28) | 38 | 2.09 | .038 |
| Angola | 481 (39) | 15 | 494 (40) | 17 | .88 | .384 |
| Argentina | 512 (30) | 24 | 511 (30) | 93 | –.04 | .972 |
| Armenia | 486 (25) | 75 | 493 (27) | 146 | 1.66 | .099 |
| Australia | 544 (40) | 26 | 542 (39) | 83 | –.25 | .804 |
| Austria | 571 (42) | 17 | 573 (43) | 142 | .22 | .829 |
| Belgium | 529 (41) | 23 | 520 (32) | 42 | –.98 | .333 |
| Bolivia | 498 (45) | 6 | 505 (34) | 25 | .45 | .656 |
| Brazil | 510 (38) | 87 | 507 (35) | 319 | –.80 | .423 |
| Bulgaria | 527 (40) | 31 | 522 (34) | 260 | –.78 | .437 |
| Burundi | 477 (22) | 45 | 483 (22) | 29 | 1.03 | .304 |
| Cameroon | 486 (29) | 36 | 483 (29) | 21 | –.39 | .701 |
| Canada | 541 (39) | 20 | 543 (42) | 98 | .26 | .792 |
| Cape Verde | 499 (31) | 16 | 490 (27) | 19 | –.93 | .361 |
| Chile | 489 (21) | 13 | 507 (34) | 58 | 1.82 | .073 |
| China | 484 (31) | 75 | 493 (26) | 268 | 2.43 | .016 |
| Colombia | 506 (38) | 55 | 497 (29) | 193 | –1.61 | .112 |
| Congo, Dem. Rep. | 474 (19) | 32 | 488 (21) | 8 | 1.79 | .082 |
| Congo, Rep. | 483 (24) | 27 | 494 (37) | 9 | 1.02 | .314 |
| Costa Rica | 503 (13) | 3 | 495 (22) | 17 | –.60 | .559 |
| Croatia | 511 (33) | 137 | 512 (31) | 225 | .32 | .749 |
| Cuba | 499 (31) | 22 | 496 (31) | 34 | –.38 | .707 |
| Czech Rep. | 553 (44) | 18 | 533 (37) | 299 | –2.19 | .029 |
| Denmark | 557 (32) | 17 | 557 (37) | 107 | .06 | .951 |
| Dominican Rep. | 482 (11) | 4 | 489 (18) | 29 | .74 | .463 |
| Ecuador | 500 (31) | 14 | 501 (29) | 42 | –.08 | .940 |
| Egypt | 486 (26) | 164 | 487 (27) | 52 | .26 | .796 |
| Eritrea | 489 (35) | 16 | 472 (21) | 10 | –1.35 | .180 |
| Estonia | 563 (0) | 1 | 546 (36) | 30 | –.47 | .643 |
| Ethiopia | 482 (27) | 61 | 482 (37) | 18 | .09 | .925 |
| Finland | 562 (46) | 17 | 555 (43) | 146 | –.61 | .541 |
| France | 527 (41) | 103 | 522 (38) | 484 | –1.26 | .209 |
| Georgia | 501 (23) | 12 | 495 (35) | 27 | –.57 | .571 |
| Germany | 571 (44) | 353 | 567 (45) | 1557 | –1.21 | .227 |
| Greece | 514 (34) | 69 | 514 (34) | 77 | .03 | .976 |
| Guatemala | 487 (25) | 6 | 497 (23) | 17 | .94 | .357 |
| Hong Kong | 498 (46) | 8 | 488 (23) | 40 | –.62 | .554 |
| Hungary | 527 (49) | 33 | 531 (40) | 383 | .42 | .618 |
| Iceland | 526 (24) | 9 | 536 (31) | 19 | .78 | .445 |
| India | 499 (36) | 23 | 505 (27) | 54 | .72 | .475 |
| Indonesia | 485 (27) | 212 | 492 (27) | 696 | 2.93 | .003 |
| Iran | 485 (27) | 779 | 485 (26) | 676 | .23 | .815 |

| Country | Males Mean (SD) | N | Females Mean (SD) | N | T-test | p-value |
| --- | --- | --- | --- | --- | --- | --- |
| Iraq | 482 (25) | 921 | 480 (24) | 373 | –1.26 | .210 |
| Ireland | 530 (34) | 22 | 535 (35) | 86 | .53 | .595 |
| Italy | 519 (32) | 133 | 524 (37) | 253 | 1.30 | .196 |
| Japan | 483 (20) | 11 | 496 (29) | 170 | 2.06 | .060 |
| Jordan | 492 (26) | 16 | 491 (23) | 9 | .11 | .914 |
| Korea Rep. | 483 (15) | 3 | 497 (26) | 32 | .94 | .357 |
| Kuwait | 493 (32) | 15 | 492 (34) | 12 | –.05 | .963 |
| Latvia | 483 (0) | 1 | 527 (29) | 27 | 1.50 | .145 |
| Lebanon | 490 (27) | 28 | 493 (31) | 21 | .42 | .676 |
| Liberia | 479 (21) | 18 | 476 (23) | 4 | –.27 | .794 |
| Lithuania | 515 (20) | 4 | 521 (35) | 80 | .32 | .750 |
| Malaysia | 515 (35) | 9 | 500 (23) | 49 | –1.62 | .111 |
| Mexico | 489 (29) | 23 | 505 (31) | 144 | 2.43 | .016 |
| Morocco | 481 (26) | 1582 | 486 (26) | 717 | 4.33 | <.001 |
| Netherlands | 494 (34) | 146 | 499 (42) | 135 | 1.08 | .283 |
| New Zealand | 534 (39) | 11 | 551 (46) | 29 | 1.08 | .288 |
| Nigeria | 494 (32) | 58 | 479 (21) | 21 | –1.91 | .060 |
| Norway | 552 (36) | 19 | 547 (40) | 91 | –.53 | .600 |
| Peru | 501 (35) | 33 | 502 (29) | 155 | –.04 | .966 |
| Philippines | 492 (12) | 14 | 484 (22) | 186 | –1.40 | .164 |
| Poland | 526 (34) | 75 | 520 (35) | 1416 | –1.65 | .100 |
| Portugal | 516 (49) | 20 | 519 (38) | 93 | .31 | .757 |
| Romania | 520 (36) | 67 | 518 (34) | 450 | –.50 | .620 |
| Russian Federation | 517 (38) | 219 | 515 (35) | 1386 | –.58 | .561 |
| Rwanda | 474 (19) | 63 | 479 (19) | 52 | 1.26 | .209 |
| Serbia | 508 (32) | 679 | 508 (34) | 1037 | .05 | .960 |
| Singapore | 506 (42) | 3 | 530 (43) | 25 | .91 | .371 |
| Somalia | 482 (28) | 219 | 479 (25) | 43 | .55 | .584 |
| South Africa | 554 (43) | 48 | 541 (34) | 93 | –1.90 | .060 |
| Spain | 517 (35) | 86 | 518 (37) | 454 | .25 | .804 |
| Sri Lanka | 486 (26) | 31 | 474 (18) | 24 | –1.90 | .063 |
| Sudan | 477 (23) | 202 | 483 (28) | 47 | 1.39 | .167 |
| Sweden | 569 (47) | 24 | 571 (44) | 145 | .19 | .853 |
| Switzerland | 570 (30) | 34 | 579 (39) | 107 | 1.25 | .212 |
| Syria | 488 (26) | 109 | 485 (29) | 52 | –.60 | .547 |
| Thailand | 488 (25) | 15 | 482 (21) | 116 | –.93 | .356 |
| Tunisia | 490 (32) | 56 | 488 (34) | 29 | –.40 | .694 |
| Turkey | 480 (28) | 947 | 484 (29) | 573 | 2.50 | .013 |
| Ukraine | 524 (38) | 9 | 510 (32) | 112 | –1.26 | .210 |
| United Kingdom | 540 (412) | 217 | 539 (42) | 429 | –.24 | .814 |
| United States | 536 (44) | 138 | 539 (42) | 340 | .70 | .483 |
| Uruguay | 541 (57) | 5 | 535 (42) | 20 | –.27 | .791 |
| Venezuela | 501 (37) | 16 | 495 (27) | 88 | –.85 | .398 |
| Vietnam | 492 (35) | 25 | 479 (21) | 77 | –2.26 | .026 |

**S4 Appendix 4.** Mean listening scores (SD) of male and female learners, T-tests and p-values.
